# Unveiling the hidden dangers: enteropathogens carried by flies in Pudong New Area

**DOI:** 10.1186/s12879-024-09448-0

**Published:** 2024-06-07

**Authors:** Chen Lin, Jun Liu, Yongting Yuan, Siyu Yu, Lei Feng, Yingpei Gu, Xinchen Lu, Jingyi Liu, Huihui Li, Chenxi Hu, Hongxia Liu, Hanzhao Liu

**Affiliations:** 1 Pudong New Area Center for Disease Control and Prevention, Shanghai, 200136 China; 2 Fudan University Pudong Institute of Preventive Medicine, Shanghai, 200136 China; 3 Shanghai Center for Disease Control and Prevention, Shanghai, 200336 China

**Keywords:** Enteropathogens, Fly, *Musca domestica*, *Lucilia Sericata*, *Boettcherisca Peregrina*, Bacteria, Viruses, Parasites

## Abstract

**Background:**

Flies are acknowledged as vectors of diseases transmitted through mechanical means and represent a significant risk to human health. The study aimed to determine the prevalence of enteropathogens carried by flies in Pudong New Area to inform strategies for preventing and controlling flies.

**Methods:**

Samples were collected from various locations in the area using cage trapping techniques between April and November 2021, encompassing various habitats such as parks, residential areas, restaurants, and farmers’ markets. The main fly species were identified using cryomicrography and taxonomic enumeration, with 20 samples per tube collected from different habitats. Twenty-five enteropathogens were screened using GI_Trial v3 TaqMan^TM^ microbial arrays.

**Results:**

A total of 3,875 flies were collected from 6,400 placements, resulting in an average fly density of 0.61 flies per cage. *M. domestica* were the most common species at 39.85%, followed by *L. sericata* at 16.57% and *B. peregrina* at 13.14%. Out of 189 samples, 93 tested positive for enteropathogens, with nine different pathogens being found. 12.70% of samples exclusively had parasites, a higher percentage than those with only bacteria or viruses. The study found that *M. domestica* had fewer enteropathogens than *L. sericata* and *B. peregrina*, which primarily harbored *B. hominis* instead of bacteria and viruses such as *E. coli*, Astrovirus, and Sapovirus. During spring testing, all three fly species exhibited low rates of detecting enteropathogens. *M. domestica* were found in residential areas with the highest number of pathogen species, totaling six. In contrast, *L. sericata* and *B. peregrina* were identified in farmers’ markets with the highest number of pathogen species, totaling six and seven, respectively.

**Conclusions:**

Flies have the potential to serve as vectors for the transmission of enteropathogens, thereby posing a substantial risk to public health.

**Supplementary Information:**

The online version contains supplementary material available at 10.1186/s12879-024-09448-0.

## Introduction

Intestinal infectious diseases (IIDs) are a group of diseases caused by bacteria, viruses, parasites and other pathogens that are transmitted through the digestive tract, with fever and diarrhea as the main symptoms [1]. IIDs are a significant global public health concern, exerting a substantial impact on the public health domain and serving as a prominent contributor to the increased mortality rate among children under the age of five [2, 3]. In China, IIDs remain a predominant concern in the realm of infectious diseases, resulting in significant economic and health burdens [4–6]. Scientific investigations in the late 19th century conclusively established the involvement of Diptera as vectors in the dissemination of enteropathogens [7]. During the 20th century, the rise in cases of enteric infections prompted Western countries to prioritize research on Diptera, particularly species with valves [8–11]. Tufts University Medical Center in the United States conducted a study on fecal samples from individuals with diarrhea in urban and rural households in Vellore, India, and examined nearby flies to investigate the influence of environmental factors on the spread of IIDs [12]. The high frequency of human mobility and trade activities in the Pudong New Area poses a substantial risk for the transmission of IIDs. During the period of 2013 to 2017, there was a notable persistence of high positive detection rates of enteropathogens in Pudong New Area, with Norovirus, Rotavirus, and *Escherichia coli*(*E. coli*) emerging as predominant pathogens exhibiting distinct seasonal epidemiological patterns [6]. Against this background, it is crucial to study the transmission routes of enteric infectious diseases and the impact of pathogens on public health.

Diptera, specifically flies, are considered significant environmental health pests due to their ability to transmit pathogens through primarily mechanical means, with some instances of biological transmission [13, 14]. This insect group possesses abundant body and foot hairs that facilitate the transportation of various microorganisms, including bacteria such as *E. coli*, Staphylococcus aureus, Klebsiella spp, Bacillus spp, and Acinetobacter spp [15–20], as well as viruses like Adenoviruses and Rotaviruses [15, 21], and a diverse array of parasites such as *Cryptosporidium* [21–24]. *E. coli* is a key indicator of potential contamination in water and food sources, as well as a direct threat to human health through pathogenic strains like ETEC, EHEC, EIEC, and EPEC causing various illnesses including diarrhea [25]. Some strains of *E. coli* have virulence factors like toxin and antibiotic resistance genes, making treatment difficult. Non-pathogenic strains in the intestines can help with normal functions, but can become carriers of virulence factors when transmitted by flies. *Cryptosporidium*, a common parasite found in vertebrates, can cause a diarrheal illness called cryptosporidiosis. If the host’s immune system is weak, the disease can become chronic and life-threatening. Cryptosporidium tyzzeri, known for infecting a limited range of hosts, is also capable of spreading to humans [26]. The conducive hot and rainy climate, high population density, significant domestic waste generation, and diverse industrial structure of Pudong New Area create an optimal breeding environment for flies [27]. However, research on flies in Shanghai is currently limited to density monitoring and ecological studies. Several studies conducted by the Centers for Disease Control and Prevention (CDC) in China have revealed a strong correlation between fly populations and the prevalence of enteric infections and bacillary dysentery [28–30]. As environmental pollution and climate change continue to worsen, the potential impact of flies on human health is expected to escalate. Consequently, it is imperative to enhance surveillance and research efforts on fly-borne pathogens, in conjunction with exploring more efficient prevention and control measures, alongside density monitoring and ecological studies.

Within the subfamily Cyclostomatidae, several families hold significant importance in relation to human health, including Muscidae, Calliphoridae, Sarcophagidae, and Drosophilidae, totaling approximately 305 species [31]. In an ecosystem, the dominant fly species is characterized by having the largest number of individuals, the widest distribution, and the most significant impact on the environment [32]. In vector organism monitoring conducted in China and Shanghai, the species with the highest densities are *Musca domestica*(*M. domestica*), *Lucilia sericata*(*L. sericata*) and *Boettcherisca peregrina*(*B. peregrina*) [33, 34]. In addition to transmission by Diptera, environmental factors and seasonal variations play an important role in the transmission of enteric pathogens. Furthermore, the study seeks to establish potential associations between these pathogens and human enteropathogens, with the ultimate goal of providing a scientific foundation for the early warning and prediction of IIDs.

## Materials and methods

### Study area and trapping of fly species

The population of Pudong New Area is estimated to be 5,767,700 in 2021. The climate in the region is characterized by average monthly temperatures ranging from 13.5 to 28.6 °C between April and November, along with an average monthly relative humidity of 72-85%. The study categorized seasons as winter (December-February), spring (March-May), summer (June-August), and autumn (September-November) [35].

**Fig. 1:**
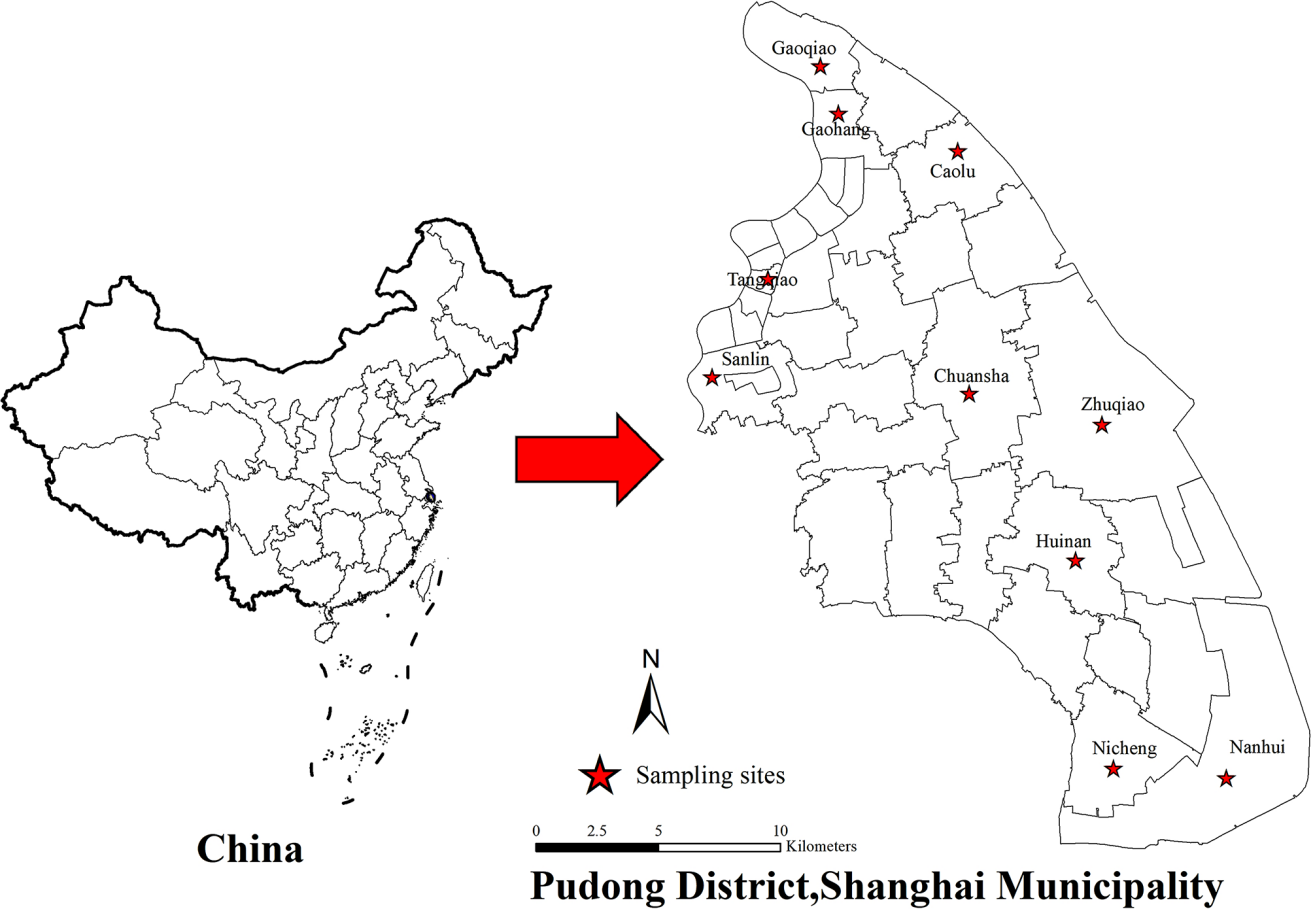
Map showing of monitoring town in Pudong New Area Shanghai, in Eastern China

Fly species were systematically collected on a monthly basis from April to November 2021. Data collection is carried out by skilled collectors on 10 specified streets, encompassing diverse habitats including parks, residential areas, farmers’ markets, and restaurants. These streets are situated in various regions of Pudong, including the eastern (Tangqiao, Sanlin), southern (Nicheng, Nanhui), western (Zhuqiao, Huinan), northern (Gaoqiao, Gaohang), and central (Caolu, Chuansha) parts(Fig. 1). The monitoring approach employed was in accordance with the cage trapping method [33]utilized in the National Vector Monitoring Implementation Programme for fly surveillance. A conical fly trap cage, measuring 40 cm in height and Φ25 cm in diameter, was utilized in conjunction with a conical core measuring 35 cm in height and featuring a top opening of Φ2 cm.The bait employed consisted of a mixture of brown sugar and vinegar (25 g each) dissolved in 25 ml of water. The object was positioned at 09:00 for the duration of the monitoring period and subsequently recovered by 09:00 on the subsequent day. Over the course of the survey, specimens of diarrheal diseases will be collected from 10 monitoring point hospitals, such as Shanghai East Hospital, Renji Hospital, Sixth People’s Hospital, Seventh People’s Hospital, Pudong Hospital, Pudong New District People’s Hospital, Gongli Hospital, NiCheng Community Hospital, Zhoupu Hospital and Yang Si Hospital. These specimens will then be sent to the Pudong New Area CDC for microbiological testing.

### Fly identification and sample testing

The collector promptly brought the fly specimens to the laboratory and subjected them to freeze sterilization before transferring them to disposable petri dishes after a 30-minute interval. The petri dishes were then arranged in rows on ice.Following the protocol outlined in “Handbook of Classification and Identification of Major Disease Vectors,” edited by Zhou Minghao [36], we individually identified the morphological characteristics and quantities of the fly species using a stereomicroscope. Subsequently, we determined the fly densities and species compositions in order to identify the three dominant fly species. To facilitate further analysis, 2 ml grinding tubes were prepared with a solution of Hank’s buffer, Proteinase K, and grinding beads, all pre-cooled to -20 °C. Identical fly species collected from the same habitat were grouped together in a grinding tube as a single sample, with a maximum of 20 flies per sample [12].The tubes were securely capped and placed in an aluminum adapter, then ground for 0.5 min at room temperature (55 Hz) using a high-throughput tissue grinder. Subsequently, the tubes were centrifuged for 4 min using an ultra-cold high-speed centrifuge (centrifugation radius 8.4 cm, 2,000 rpm, 4 °C). Following centrifugation, 200 µl of supernatant was pipetted for nucleic acid extraction, while the remaining sample was reserved for further analysis.

Nucleic acids were isolated using Nucleic Acid Extraction Reagent with a fully automated nucleic acid extractor. The isolated nucleic acids were then mixed with premix and used on GI_Trial v3 TaqMan^TM^ microbial arrays. Real-time fluorescence quantitative reverse transcription polymerase chain reaction (RT-PCR) assays were conducted employing a Quant Studio 7 Fluorescence Quantitative PCR Instrument. The resulting amplification curve morphology, Amp Score, Cq value, and corresponding Cq confidence were evaluated in accordance with the manufacturer’s guidelines for qualitative identification of 25 enteropathogens. The GI Microfluidic Chip V3 Premix is capable of detecting a total of 13 bacteria, including *E. coli*, *Vibrio parahaemolyticus* (*V. parahaemolyticus*), *Vibrio vulnificus*, *Vibrio cholerae*, *Aeromonas hydrophila*, *Plesiomonas shigelloides*, *Yersinia enterocolitica*, *Campylobacter jejuni*, *Campylobacter coli*, *Campylobacter upsaliensis*, *Clostridium difficile*, *Salmonella*, and *Shigella*. It can also detect 6 viruses, namely Norovirus, Astrovirus, Sapovirus, Adenovirus, Rotavirus, and Parechovirus. In addition, six parasites were identified in the study, including *Blastocystis hominis* (*B. hominis*), *Cryptosporidium*, *Dientamoeba fragilis*, *Entamoeba histolytica*, *Cyclospora cayetanensis*, and *Giardia lamblia*. The findings were confirmed using fluorescence quantitative PCR with designed primers, which provided accurate and reliable results.

### Calculation of indicators

Adult fly density in cages = total number of flies caught in cages divided by the number of cages.

One tube is capable of detecting a range of 0 to M viruses, bacteria, or parasites. The presence of a pathogen is indicated by the detection of one positive tube among a total number of tubes tested. The rate of single pathogen detection is calculated by dividing the number of tubes testing positive for a single pathogen by the total number of tubes tested, denoted as M/N, where M represents the number of pathogen-positive test tubes and N represents the total number of tubes tested.

### Statistical analysis

Data were compiled using Excel 2019 software and subsequently analyzed using SPSS 19.0 software through the Pearson χ2 test, employing a two-sided test with a significance level of *P* < 0.05.

## Results

Fly species and density.

Between April and November 2021, a total of 6,400 cages were utilized to capture 3,875 fly species, resulting in an average density of 0.61 fly species per cage. The composition of fly species revealed that *M. domestica* constituted the largest proportion at 39.85%, with a density of 0.24 per cage. Following *M. domestica*, *L. sericata* accounted for 16.57% and *B. peregrina* accounted for 13.14%, with densities of 0.10 and 0.08 per cage, respectively (Table 1, and Database: Supplementary Information 1).

**Table 1:** displays the density and species composition of fly species

Species	Cages	Number	Densities(fly species/cage )	Component ratio(%)
*M. domestica*	6400	1544	0.24	39.85
*L. sericata*	6400	642	0.10	16.57
*B. peregrina*	6400	509	0.08	13.14
Other species of Sarcophaga	6400	488	0.08	12.59
Other species of Lucilia	6400	287	0.04	7.41
*C. megacephala*	6400	196	0.03	5.06
*M. stabulans*	6400	111	0.02	2.86
*M. sorbens*	6400	52	0.01	1.34
*F. canicularis*	6400	26	0.00	0.67
*F. prisca*	6400	20	0.00	0.52
Total	6400	3875	0.61	100.00

### The transportation of enteropathogens by fly species

**Fig. 2:**
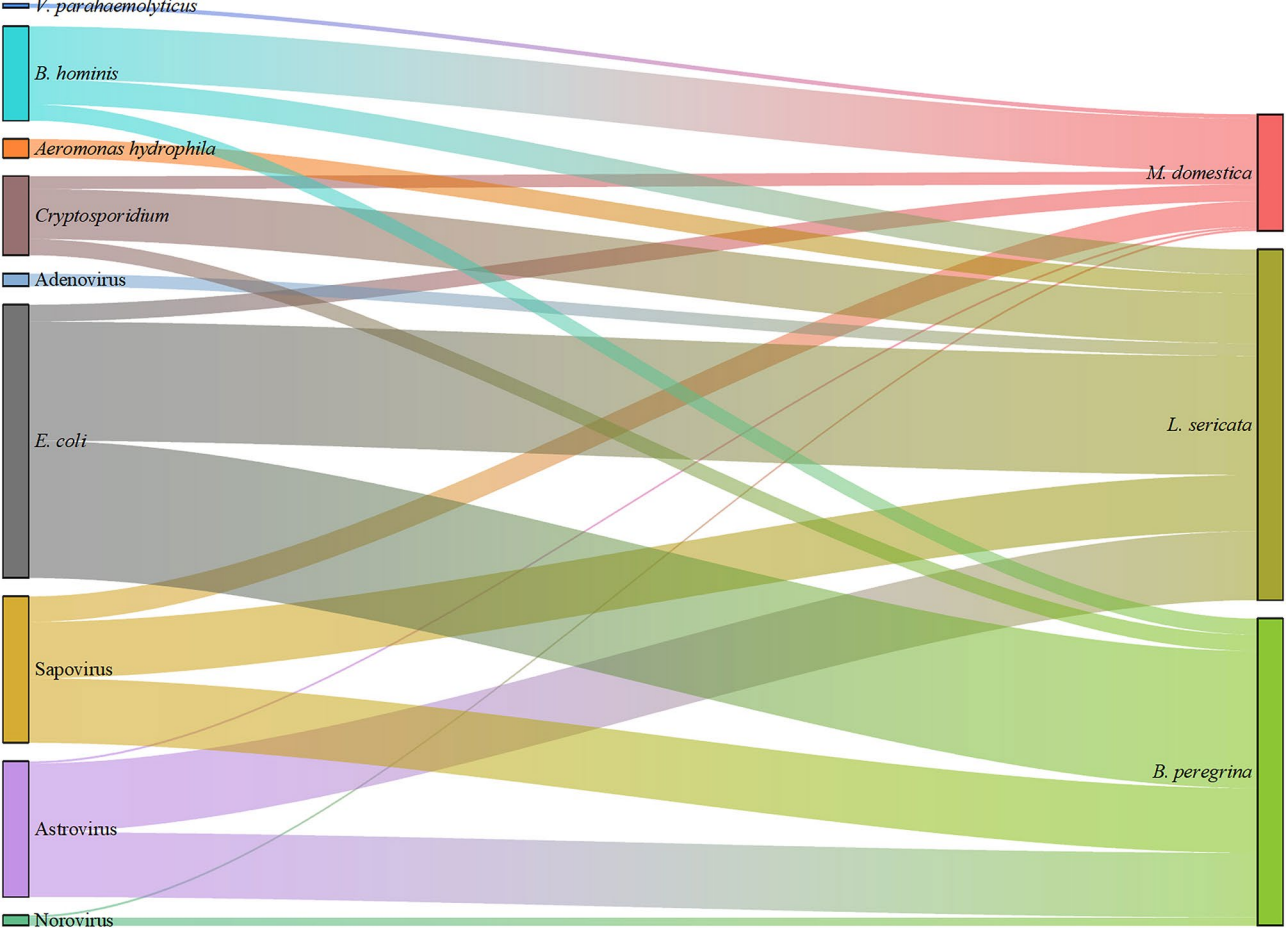
Displays the enteropathogens identified in fly species

A total of 189 samples were collected from three species of flies, with 93 samples testing positive for enteropathogens. Among the infected fly species, 26.88% were found to carry only parasites, a higher proportion compared to fly species carrying only bacteria and viruses. Additionally, 29.04% of the infected fly species carried two major types of enteropathogens, while 5.38% carried all types of enteropathogens. The detection rate of enteropathogens was found to be significantly lower (*P* < 0.05) compared to that of *L. sericata* and *B. peregrina*. The enteropathogens carried by *M. domestica* primarily consisted of parasites, particularly *B. hominis*. In contrast, *L. sericata* and *B. peregrina* harbored enteropathogens such as *E. coli*, Astrovirus, and Sapovirus to a lesser extent (Table 2; Fig. 2, and Database: Supplementary Information 2).

**Table 2:** Displays the different types of enteropathogens found in fly species

Category	Samples of fly species *n*(%)		*P*	Types of enteropathogens *n*(%)				
	*N*	Positive		Only bacteria	Only viruses	Only parasites	carried 2 types	carried 3 types
All	189	93(49.21)		18(19.35)	18(19.35)	25(26.88)	27(29.04)	5(5.38)
Species								
*M.domestica*	118	44(37.29)	<0.05	4	10	23	7	0
*L. sericata*	40	27(67.50)		6	5	0	11	5
*B. peregrina*	31	22(70.97)		6	3	2	10	1
Seasons								
spring	39	17(43.59)	>0.05	6	4	3	4	0
summer	77	38(49.35)		7	10	7	10	4
autumn	73	38(52.05)		4	4	15	13	2

### Fly species carry and spread enteropathogens throughout the year

**Fig. 3:**
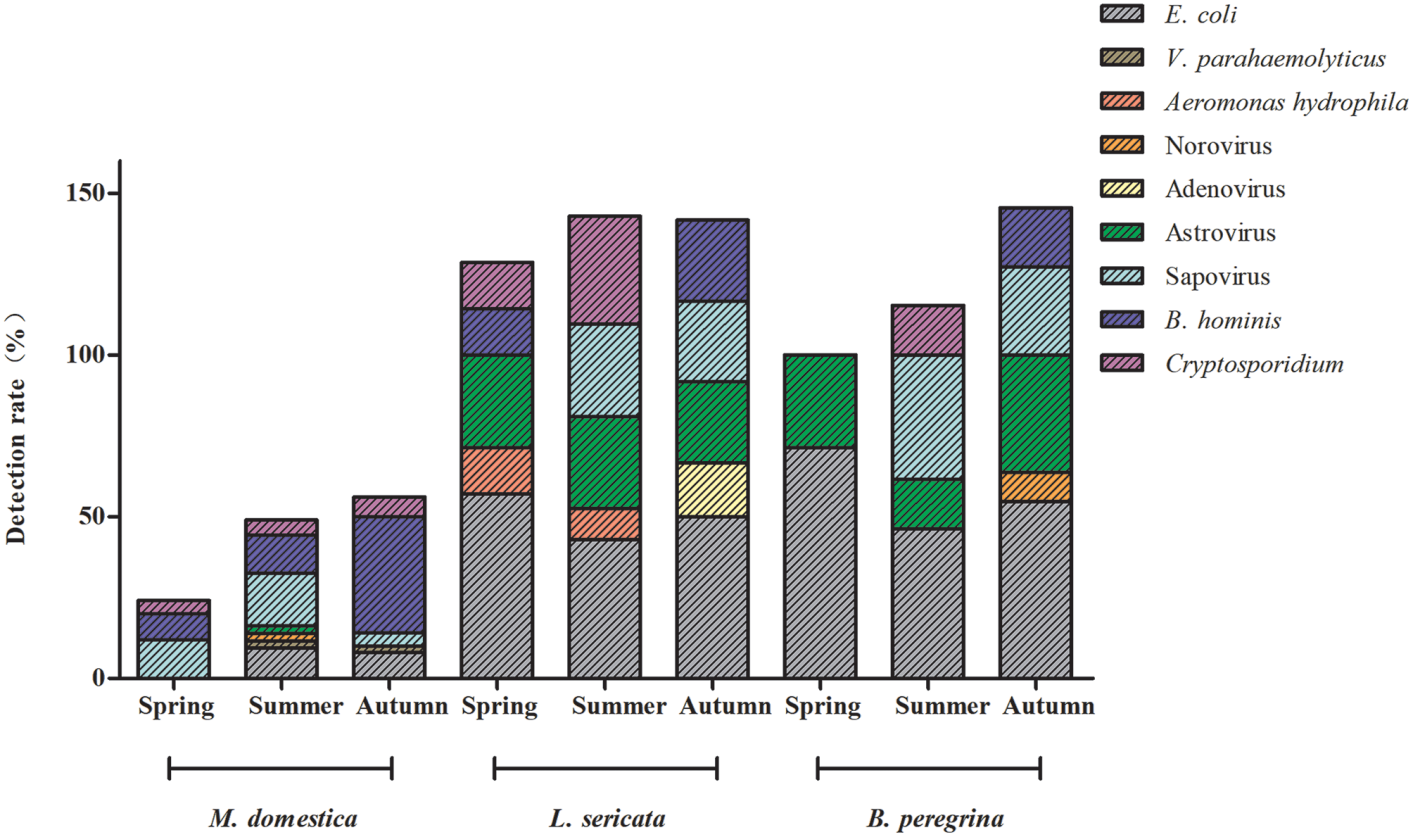
Shows the identification of enteropathogens carried by fly species throughout various seasons

During the spring season, all three fly species exhibited the lowest rates and varieties of enteropathogens. However, *Cryptosporidium* was present in all fly species during the summer season, while *B. hominis* was found at the highest rate in the autumn. *M. domestica* samples showed the highest number of pathogenic species in the summer, including Norovirus and Astrovirus. *L. sericata* samples revealed the presence of *Aeromonas hydrophila* in both spring and summer, and Adenovirus in autumn. *B. peregrina* showed a high diversity of enteropathogens during the autumn season, with Norovirus being detected (Fig. 3).

### Fly species carry enteropathogens in various habitats

**Fig. 4:**
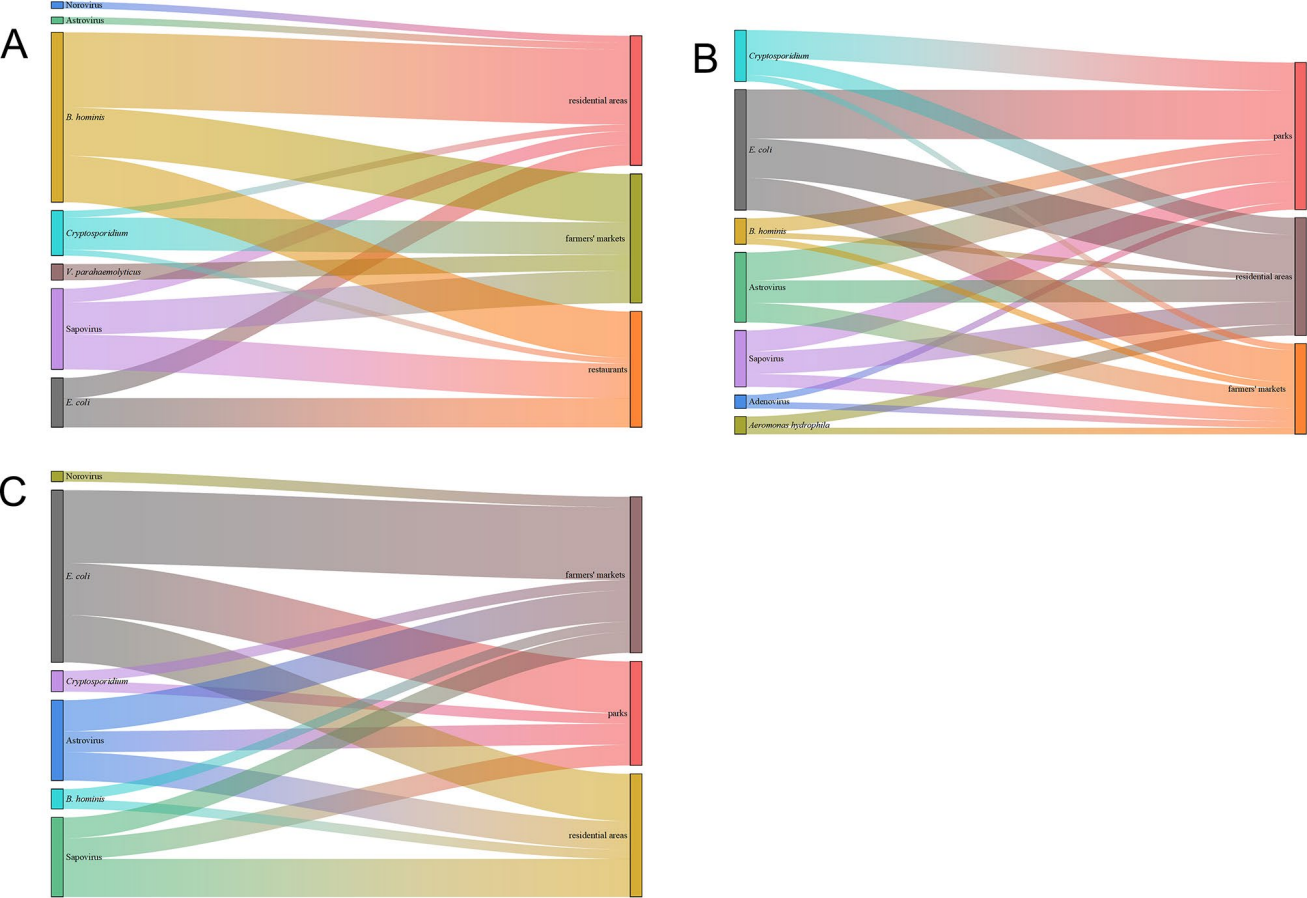
Illustrates the various types of enteropathogens harbored by different species of fly across various habitat types **(A)***M. domestica* transports enteropathogens across various habitat types. **(B)***L. sericata* transports enteropathogens across various habitat types. **(C)***B. peregrina* transports enteropathogens across various habitat types

*M. domestica* exhibited the lowest presence in residential areas and farmers’ markets, predominantly harboring parasites, while *L. sericata* and *B. peregrina* were found to predominantly carry bacteria and viruses such as *E. coli*, Astroviruses, and Sapovirus. Among the three habitats of *M. domestica*, *B. hominis* showed the highest rate of detection, followed by Sapovirus and *Cryptosporidium* in second and third place, respectively. Residential areas had the highest number of enteropathogens detected, with a total of six species identified, including *V. parahaemolyticus* found in farmers’ markets. Farmers’ markets had the highest number of pathogens detected, with six species of *L. sericata* and seven species of *B. peregrina*, respectively. *L. sericata* were found to carry *Aeromonas hydrophila* in both residential areas and farmers’ markets, and *B. peregrina* were found to carry Norovirus in farmers’ markets (Table 3; Fig. 4, and Database: Supplementary Information 2*).*

**Table 3:** Diversity of enteropathogens carried by fly species across different habitat types

Habitats	Samples of fly species *n*(%)		*P*	Types of pathogens *n*(%)				
	*N*	Positive		Only bacteria	Only viruses	Only parasites	carried 2 types	carried 3 types
All	189	93(49.21)		18(19.35)	18(19.35)	25(26.88)	27(29.04)	5(5.38)
Residential area								
*M. domestica*	39	15(38.46)	<0.05	1	3	9	2	0
*L. sericata*	15	10(66.67)		3	2	0	3	2
*B. peregrina*	11	8(72.73)		2	2	1	3	0
Market								
*M. domestica*	33	12(36.36)	<0.05	1	3	6	2	0
*L. sericata*	13	7(53.85)		2	1	0	3	1
*B. peregrina*	10	8(80.00)		3	0	1	3	1
All species								
Park	22	16(72.73)	>0.05	2	3	0	9	2
Residential area	65	33(50.77)		6	7	10	8	2
Farmers’ markets	56	27(48.21)		7	4	7	7	2
Restaurant	46	17(36.96)		2	4	8	3	0

### The diversity of fly species and their potential role as vectors for human enteropathogens

**Fig. 5:**
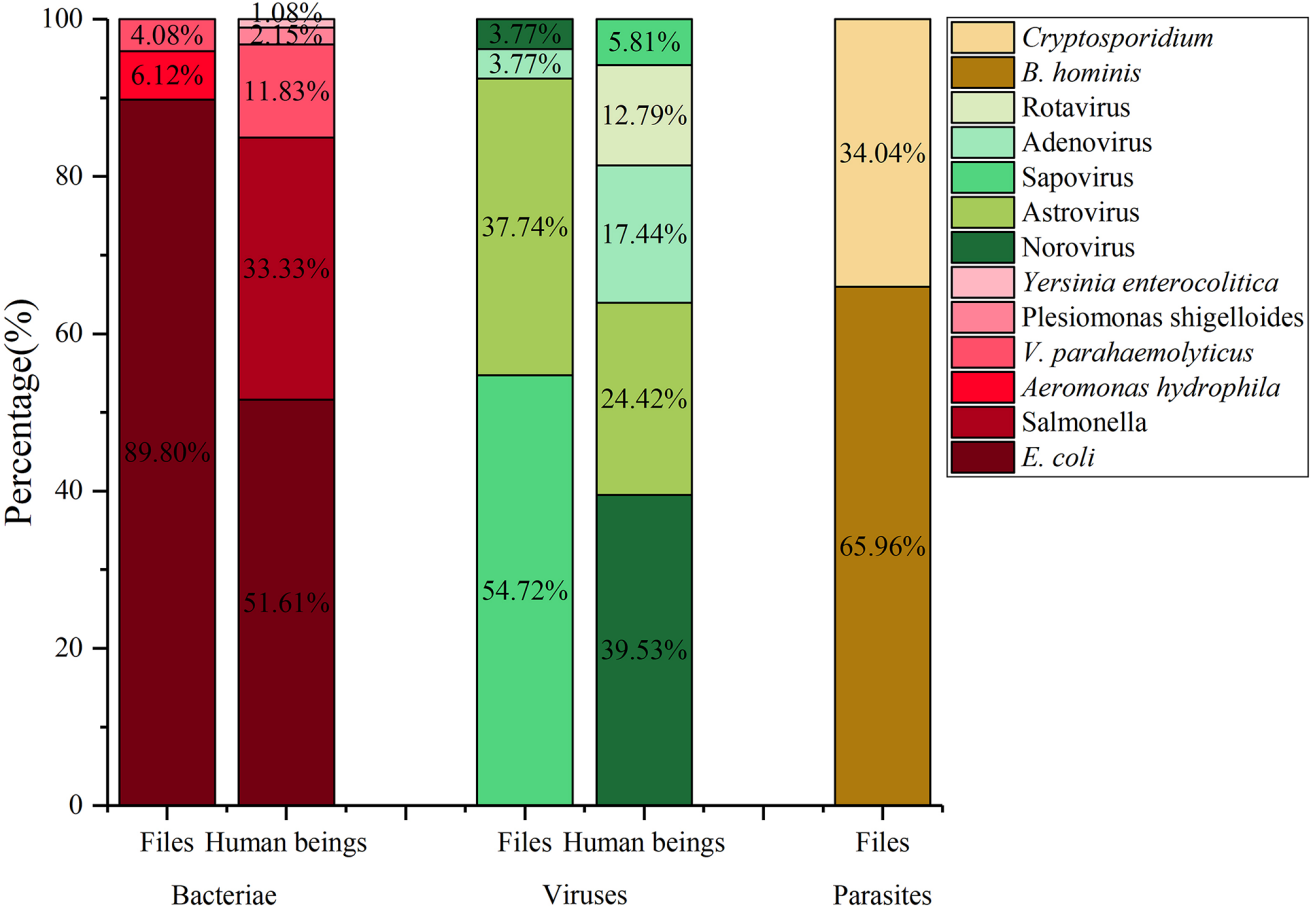
Illustrates the comparison between the detection of enteropathogens in fly species and human beings

The spectrum of enteropathogens identified in fly species included nine pathogens, with ten pathogens isolated from the comprehensive surveillance of IIDs. Among the bacteria detected in fly species, *E. coli* accounted for 89.80%, *V. parahaemolyticus* for 4.08%, and *Aeromonas hydrophila* for 6.12%. In the surveillance of human beings, *E. coli* was detected in 51.61% of cases, *Salmonella* in 33.33%, *V. parahaemolyticus* in 11.83%, *Plesiomonas shigelloides* in 2.15%, and *Yersinia enterocolitica* in 1.08%. Sapovirus demonstrated the highest frequency of isolation at 54.72%, followed by Astrovirus at 37.74%. Conversely, Norovirus was the most prevalent virus identified in human surveillance, accounting for 38.8% of cases, with Astrovirus and Adenovirus following at 24.42% and 17.44%, respectively. *B. hominis* and *Cryptosporidium* were predominantly identified in fly species, with relative frequencies of 65.96% and 34.04%, respectively. No parasites were detected during the monitoring of human diarrheal diseases (Fig. 5, and Database: Supplementary Information 3).

**Fig. 6:**
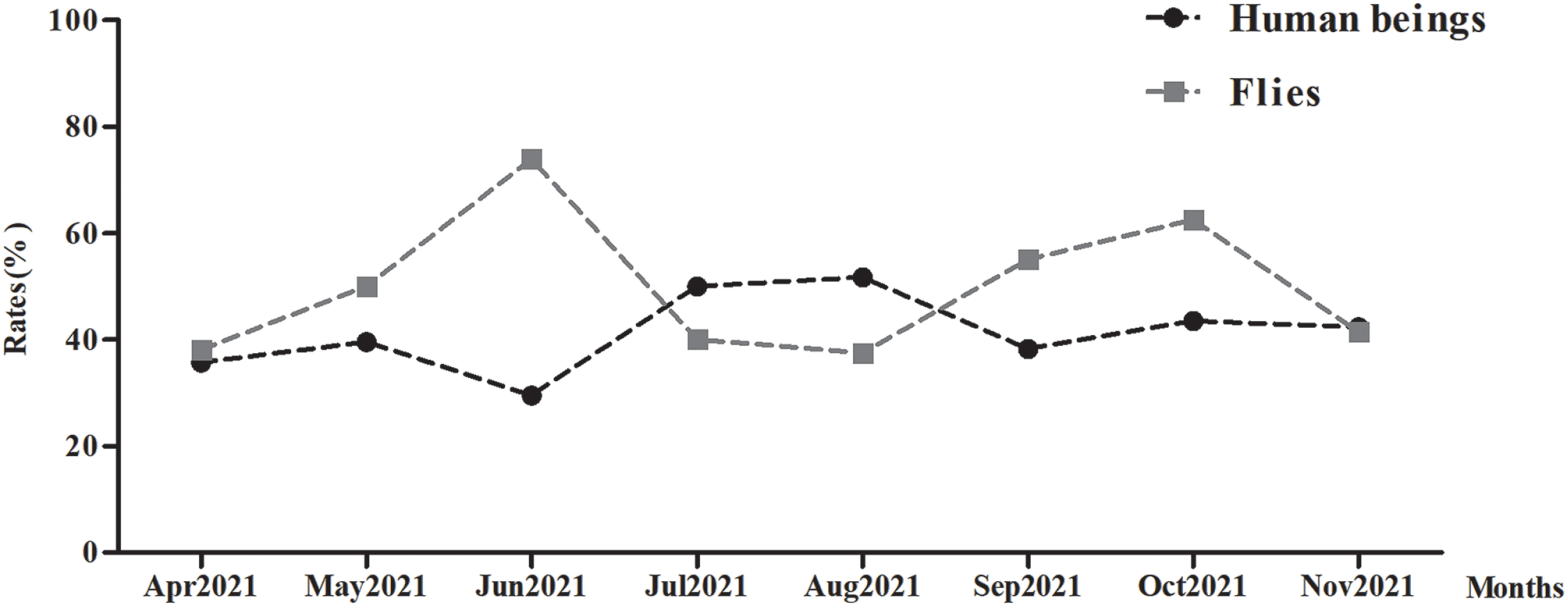
Presents a comparative analysis of the monthly distribution patterns of enteropathogens detection rates in fly species and human beings

The temporal trends of enteropathogens differed in fly species and in human beings, with peaks in detection occurring in different months. Fly species were most commonly detected in June and October, with lower detection rates in other months, while human enteropathogens peaked primarily in May, August, and October (Fig. 6, and Database: Supplementary Information 3).

## Discussion

The study results revealed the presence of up to nine pathogens in three common fly species, including three bacteria (*E. coli*, *V. parahaemolyticus*, and *Aeromonas hydrophila*), four viruses (Sapovirus, Astrovirus, Norovirus, and Adenovirus), and two protozoan parasites (*B. hominis* and *Cryptosporidium*). *E. coli*, *B. hominis*, and Sapovirus were the most common pathogens found, while highly pathogenic pathogens like *Shigella* and *Vibrio cholerae* were not detected [37]. *E. coli* was consistent with previous studies [38]. *B. hominis*, a zoonotic waterborne parasite, has been found in fecal samples from patients at Peking Union Medical College Hospital and Shanghai residents [39, 40]. These viruses have not been found in flies before, but Sapovirus and Astrovirus, which are easily spread to infants and young children, were found in all three flies. Sapovirus and Astrovirus, both foodborne viruses, are known to induce acute gastroenteritis and exhibit heightened pathogenicity in individuals with underlying chronic conditions or compromised immune systems, resulting in severe symptoms, prolonged illness, and an increased likelihood of complications [41–43]. Sapovirus was discovered in children with diarrhea at Shanghai East Hospital [44], while astrovirus was found in children with acute gastroenteritis at Fudan University’s pediatric hospital [45]. The lack of research on Sapovirus and Astrovirus in flies highlights the importance of studying flies as carriers of diseases. Norovirus has been implicated in fatalities among the elderly, as well as contributing to morbidity and mortality in young children, immunocompromised individuals, and the elderly. Furthermore, Norovirus infections incur healthcare expenses and diminish productivity [46, 47]. Rotavirus is the primary etiological agent of severe gastroenteritis in elderly individuals and children on a global scale [48].

Seasonal changes affect the detection rate and diversity of enteropathogens in Diptera, with lower levels observed in the spring due to less favorable conditions for their proliferation and transmission. This finding contrasts with the results reported in Dutta’s study [38]. Furthermore, the decrease in food remnants post-spring likely impeded the formation of potential sources of transmission and breeding grounds for pathogens.This phenomenon may be attributed to precipitation events that led to the runoff of animal feces containing *Cryptosporidium* oocysts into the water source. Autumn weather changes may have made conditions better for *B. hominis* in flies, increasing its presence. In contrast, summer samples of *M. domestica* showed different pathogens than those found in humans by Wang et al [6]. *L. sericata* found *Aeromonas hydrophila* in water during warmer months, which could increase with higher temperatures. Of particular concern are the heightened temperatures and increased precipitation characteristic of summer [49], which may exacerbate environmental sanitation issues due to heightened fly activity.

Given the diverse feeding habits of *M. domestica*, which encompass a broad spectrum of organic materials such as refuse, waste, and decaying food, coupled with their frequent presence in human environments, it is plausible that their extensive contact with human food sources and water reservoirs has facilitated their role as primary vectors for the transmission of the waterborne parasites *B. hominis* and *Cryptosporidium* [50]. The prevalence of a wide range of enteropathogens in *M. domestica* is most pronounced in regions with high residential density, a phenomenon that can be linked to the accumulation of domestic waste resulting from dense population concentrations. The buildup of waste attracts *M. domestica* and promotes the spread of disease. Proper waste disposal and water cleanliness can reduce the risk of disease transmission. The main pathogens found at farmers’ markets are *L. sericata* and *B. peregrina* due to unsanitary conditions and abundant food waste and animal carcasses. This increases the risk of disease transmission, making it crucial to improve cleanliness at these markets to prevent the spread of illnesses.

Norovirus was identified in *M. domestica* captured in residential areas and in *B. peregrina* captured in farmers’ markets, a discovery that aligns with previous research by Stefan [21]. This virus is currently a leading cause of infectious diarrhea in the Pudong New Area of Shanghai [51], and the high population density in the area may have facilitated the transmission of the pathogen, potentially leading to an increased diversity of pathogens carried by the flies. It is believed that *B. peregrina* have a higher ability to spread pathogens from food and waste in markets due to their active behavior and feeding habits. Contaminated food in markets helps *B. peregrina* multiply. Limited research has been done on how flies transmit *V. parahaemolyticus*, *Aeromonas hydrophila*, and Adenovirus. *V. parahaemolyticus*, a marine pathogen, has been found in flies at farmers’ markets and can cause foodborne illness with symptoms like abdominal pain, vomiting, and diarrhea. This pathogen is commonly found in seafood and thrives in environments where food can spoil. To prevent its spread, it is important to improve hygiene practices in farmers’ markets. The presence of *Aeromonas hydrophila* in *L. sericata* collected from residential areas and farmers’ markets supports previous research findings [38]. This bacterium is a major cause of infectious disease outbreaks in freshwater aquaculture fish in China. Monitoring *L. sericata* can help understand their role in spreading *Aeromonas hydrophila*. This finding is important for preventing and managing fish diseases, highlighting the need to control *L. sericata* in residential areas and markets. Additionally, the study identified the presence of Adenovirus in *L. sericata* captured in parks and farmers’ markets, a pathogen known to induce acute gastroenteritis characterized by abdominal pain and diarrhea in children under the age of 4. This study posits that *L. sericata* may serve as potential carriers and vectors of Adenovirus, thus presenting a potential health risk to children.

This study is constrained by several limitations. Specifically, it focused solely on enteropathogens carried by major fly species, neglecting the broader spectrum of fly species and their associated enteropathogens in natural environments. Additionally, as the study only focused on the four habitat types described, it does not provide a comprehensive understanding of fly-carrying enteropathogens in the Pudong New Area. In future research, we plan to conduct experiments on flies that carry pathogens within specific habitats. We will analyze the food sources they have come into contact with and the environments they have lived in, aiming to identify cases of direct contamination in both food and the surroundings.

## Conclusions

Utilizing high-throughput polymerase chain reaction (PCR) technology, we conducted a novel study detecting human enteropathogens in wild-caught flies, revealing their potential as carriers and transmitters of various enteropathogens such as bacteria, viruses, and parasites. This finding underscores the significant role that flies may play in the spread of foodborne illnesses.Consequently, there is a pressing necessity to enhance existing environmental hygiene protocols to reduce fly exposure to unsanitary conditions, thereby impeding the transmission of pathogens.

### Electronic supplementary material

Below is the link to the electronic supplementary material.

Supplementary Material 1

Supplementary Material 2

Supplementary Material 3

## Data Availability

The online version contains supplementary material available at https://doi.org/10.7910/DVN/UYTNQW.

## Abbreviations

M.Domestica: Musca domestica
L. sericata: Lucilia sericata
B. peregrina: Boettcherisca peregrine
B. hominis: Blastocystis hominis
E. coli: Escherichia coli
V. parahaemolyticus: Vibrio parahaemolyticus
IIDs: Intestinal infectious diseases
CDC: Center for Disease Control and Prevention
PCR: Polymerase chain reaction
RT-PCR: Real-time fluorescence quantitative reverse transcription polymerase chain reaction
HUS: Heomlytic uremic syndrome

## References

[1] Zhu B, Mao Y (2018). Prevalence and spatial-temporal clustering of typical notifable intestinal infectious diseases in China, 2005–2015. Chin J Public Health.

[2] Troeger C, Colombara DV, Rao PC, Khalil IA, Brown A, Brewer TG (2018). Global disability-adjusted life-year estimates of long-term health burden and undernutrition attributable to diarrhoeal diseases in children younger than 5 years. Lancet Glob Health.

[3] Das JK, Siddiqui F, Padhani ZA, Khan MH, Jabeen S, Mirani M (2023). Health behaviors and care seeking practices for childhood diarrhea and pneumonia in a rural district of Pakistan: a qualitative study. PLoS ONE.

[4] Sang XL, Liang XC, Chen Y, Li JD, Li JG, Bai L (2014). Estimating the burden of acute gastrointestinal illness in the community in Gansu Province, northwest China, 2012–2013. BMC Public Health.

[5] Deng LL, Han YJ, Wang JL, Liu HC, Li GL, Wang DY (2023). Epidemiological characteristics of notifiable respiratory infectious diseases in Mainland China from 2010 to 2018. Int J Environ Res Public Health.

[6] Wang WQ, Liu D, Zhao B, Fu HQ, Zhang ZK, Yu JX (2020). Epidemiological and etiological surveillance on infectious diarrhea in Pudong New Area, Shanghai, 2013–2017. Zhonghua Liu Xing Bing Xue Za Zhi.

[7] Grassi G. B.I Chetognati; Engelmann, 1883.

[8] Leng PE, Wang FM, Mo JC, Zhang Z, Qiu XH, Xin Z (2015). Progress and perspective of flies control. Chin J Vector Biol Control.

[9] Hewitt C (1912). G.House-Flies and how they spread Disease.

[10] West LST, Housefly (1951). Its natural history, Medical Importance, and control.

[11] Howard LO, The House Fly Disease Carrier (1911). An accounts of its dangerous activities and of the means of destroying it.

[12] GBD 2019 Diseases and Injuries Collaborators (2020). Global burden of 369 diseases and injuries in 204 countries and territories, 1990–2019: a systematic analysis for the global burden of disease study 2019. Lancet.

[13] Tan SW, Yap KL, Lee HL (1997). Mechanical transport of rotavirus by the legs and wings of Musca domestica (Diptera: Muscidae). J Med Entomol.

[14] Sarwar M (2015). Insect vectors involving in Mechanical Transmission of Human pathogens for Serious diseases. Int J Bioinf Biomed Eng.

[15] Capone D, Adriano Z, Cumming O, Irish SR, Knee J, Nala R (2023). Urban onsite sanitation upgrades and synanthropic flies in maputo, Mozambique: effects on enteric pathogen infection risks. Environ Sci Technol.

[16] Chandrakar C, Shakya S, Patyal A, Jain A, Ali SL, Mishra OP (2022). ERIC-PCR-based molecular typing of multidrug-resistant *Escherichia coli* isolated from houseflies (*Musca domestica*) in the environment of milk and meat shops. Lett Appl Microbiol.

[17] Gioia G, Freeman J, Sipka A, Santisteban C, Wieland M, Gallardo VA (2022). Pathogens associated with houseflies from different areas within a New York State dairy. JDS Commun.

[18] Yin JH, Kelly PJ, Wang CM (2022). Flies as vectors and potential sentinels for bacterial pathogens and Antimicrobial Resistance: a review. Vet Sci.

[19] Monyama MC, Onyiche ET, Taioe MO, Nkhebenyane JS, Thekisoe OMM (2022). Bacterial pathogens identified from houseflies in different human and animal settings: a systematic review and meta-analysis. Vet Med Sci.

[20] Pohlenz TD, Zavadilova K, Ghosh A, Zurek L (2018). Prevalence of shiga-toxigenic *Escherichia coli* in house flies (Diptera: Muscidae) in an urban environment. J Med Entomol.

[21] Collinet-Adler S, Babji S, Francis M, Kattula D, Premkumar PS, Sarkar R (2015). Environmental factors associated with high fly densities and diarrhea in Vellore, India. Appl Environ Microbiol.

[22] Liu Y, Chen Y, Wang N, Qin H, Zhang L, Zhang S (2023). The global prevalence of parasites in non-biting flies as vectors: a systematic review and meta-analysis. Parasit Vectors.

[23] Patel A, Jenkins M, Rhoden K, Barnes AN (2022). A systematic review of zoonotic enteric parasites carried by flies, cockroaches, and dung beetles. Pathogens.

[24] Khamesipour F, Lankarani KB, Honarvar B, Kwenti TE (2018). A systematic review of human pathogens carried by the housefly (*Musca domestica* L). BMC Public Health.

[25] Abebe E, Gugsa G, Ahmed M. Review on Major Food-Borne Zoonotic bacterial pathogens. J Trop Med. 2020;4674235. 10.1155/2020/4674235. 10.1155/2020/4674235 PMC7341400 32684938

[26] Rasková V, Kvetonová D, Sak Bl, McEvoy J, Edwinson A, Stenger B (2013). Human cryptosporidiosis caused by Cryptosporidium tyzzeri and C. Parvum isolates presumably transmitted from wild mice. J Clin Microbiol.

[27] Xue M, Shao GC, Gu MR, Wang H (2002). Analysis of the fly density in Pudong New Area. J Med Pest Control.

[28] Liu ZW, Liu GY, Li CD (2015). Correlation analysis between the flies density fluctuation and the incidence of intestinal infectious diseases in Yingkou City. Chin J Hyg Insect Equip Dec.

[29] Zhang SF, Fu XY, Zhang HY, Wei XQ, Zhu L. The relationship between density fluctuation of dominant population of flies and related diseases in Dongcheng of Beijing. Chin J Prev Med. 2016;17(2):139–142. 10.16506/j.1009-6639. 2016,02.014.

[30] Yu XH, Xu Y, Ni CR, Chen L, Zhang HS, Hu CS (2010). Relationship beween the seasonal fluctuations in fly population and density and the incidence of intestinal in fectious diseases in Wenzhou. Dis Surveill.

[31] Chen D, Zhang RL, Liu J, Zhang Z (2016). Advances in the study of pathogens carried by flies. J Pathog Biol.

[32] Avolio ML, Forrestel EJ, Chang CC, La Pierre KJ, Burghardt KT, Smith MD (2019). Demystifying dominant species. New Phytol.

[33] Wang XS, Wu HX, Liu QY (2020). National surveillance report on flies in China,2019. Chin J Vector Biol Control.

[34] Wang JJ, Xu JQ, Zhu J, Liu HX, Leng PE (2023). An analysis of fly ecological surveillance results in Shanghai, China, 2016–2021. Chin J Vector Biol Control.

[35] Glapa-Nowak A, Szczepanik M, Kwiecień J, Szaflarska-Popławska A, Flak-Wancerz A, Iwańczak B (2020). Insolation and disease severity in paediatric inflammatory bowel disease-a multi-centre cross-sectional study. J Clin Med.

[36] Zhou MH, Chu HL (2019). Handbook of classification and identification of major disease vectors.

[37] Zhang Q, Wang L, Wang XM, Zhang F, Wang K (2016). Surveillance on flies and fly-borne pathogens at Henan land port. Chin J Rane Frontier Health Quarantine.

[38] Rahuma N, Ghenghesh KS, Ben Aissa R, Elamaari A (2005). Carriage by the housefly (*Musca domestica*) of multiple-antibiotic-resistant bacteria that are potentially pathogenic to humans, in hospital and other urban environments in Misurata, Libya. Ann Trop Med Parasitol.

[39] Wang G, Zhang F (2021). Analysis of laboratory findings of parasites in inpatients of Peking Union Medical College Hospital. Lab med.

[40] Zhang XP, Li LH, Zhu Q, Fu YH, Ma XJ, Lv S (2008). Investigation on the infection of *Blastocystis Hominis* in various populations in Shanghai, China. J Pathog Biol.

[41] Meier JL (2021). Viral Acute Gastroenteritis in Special populations. Gastroenterol Clin North Am.

[42] Brown JR, Morfopoulou S, Hubb J, Emmett WA, Ip W, Shah D (2015). Astrovirus VA1/HMO-C: an increasingly recognized neurotropic pathogen in immunocompromised patients. Clin Infect Dis.

[43] Rippl M, Burkhard-Meier A, Schonermarck U, Fischereder M (2024). Sapovirus: an emerging pathogen in kidney transplant recipients?. Infection.

[44] Gong CH, Liu F, Chen M, Sun JP, Ding GD (2018). Surveillance and analysis of the pathogenic spectrum of childhood diarrhoea in Sanlin district, Shanghai, 2015–2016. Pract Prev Med.

[45] Lu L, Zhong HQ, Xu MH, Jia R, Liu PC, Su LY (2023). Diversity of classic and novel human astrovirus in outpatient children with acute gastroenteritis in Shanghai, China. Front Microbiol.

[46] Ettayebi K, Crawford SE, Murakami K, Broughman JR, Karandikar U, Tenge VR (2016). Replication of human noroviruses in stem cell-derived human enteroids. Science.

[47] Harris JP, Edmunds W, Pebody RG, Brown DW, Lopman BA (2008). Deaths from Norovirus among the Elderly, England and Wales. Emerg Infect Dis.

[48] Agocs MM, Serhan F, Yen C, Mwenda JM, de Oliveira LH, Teleb N (2014). WHO global rotavirus surveillance network: a strategic review of the first 5 years, 2008–2012. MMWR Morb Mortal Wkly Rep.

[49] Ekdahl K, Normann B, Andersson Y (2005). Could flies explain the elusive epidemiology of campylobacteriosis?. BMC Infect Dis.

[50] Otu-Bassey IB, Efretuei GK, Mbah M. Gut Parasites of medical importance harboured by Musca domestica in Calabar, Nigeria. Trop Parasitol. 2022;12(2):99–104. https://doi.org/110.4103/tp.tp_51_21. 10.4103/tp.tp_51_21 PMC9832493 36643981

[51] Liu D, Chen Z, Yan SJ, Ye CC, Zhu WP (2021). Epidemiological characteristics of norovirus infectious diarrhea in Pudong New Area of Shanghai in 2015–2019. J Public Health Prev Med.

