# Supplementary material for: Unveiling the hidden dangers: enteropathogens carried by flies in Pudong New Area
Source: BMC Infect Dis. 2024 Jun 7;24:569. doi: 10.1186/s12879-024-09448-0 (PMC11162034; doi:10.1186/s12879-024-09448-0)
Supplement: Supplementary file 1 — Supplementary Material 1 [file 12879_2024_9448_MOESM1_ESM.docx]

| Pathogen detection status | | | | | | | | | | | | | | | | | |
| --- | --- | --- | --- | --- | --- | --- | --- | --- | --- | --- | --- | --- | --- | --- | --- | --- | --- |
| **ID** | **season** | Month | **Habitat** | **Species** | **Detection result of Sample** | **Bacteria** | **Viruses** | **Parasites** | ***E.coli*** | ***V. parahaemolyticus*** | ***Aeromonas hydrophila*** | **Norovirus** | **Adenovirus** | **Astrovirus** | **Sapovirus** | ***B.hominis*** | ***Cryptosporidium*** |
| 1 | spring | 4 | residential areas | *M. domestica* | N | N | N | N | N | N | N | N | N | N | N | N | N |
| 2 | spring | 4 | residential areas | *M. domestica* | P | N | N | P | N | N | N | N | N | N | N | P | N |
| 3 | spring | 4 | residential areas | *M. domestica* | P | N | N | P | N | N | N | N | N | N | N | P | N |
| 4 | spring | 4 | residential areas | *M. domestica* | N | N | N | N | N | N | N | N | N | N | N | N | N |
| 5 | spring | 4 | farmers' markets | *M. domestica* | N | N | N | N | N | N | N | N | N | N | N | N | N |
| 6 | spring | 4 | farmers' markets | *M. domestica* | P | N | P | N | N | N | N | N | N | N | P | N | N |
| 7 | spring | 4 | farmers' markets | *M. domestica* | N | N | N | N | N | N | N | N | N | N | N | N | N |
| 8 | spring | 4 | farmers' markets | *M. domestica* | N | N | N | N | N | N | N | N | N | N | N | N | N |
| 9 | spring | 4 | restaurant | *M. domestica* | N | N | N | N | N | N | N | N | N | N | N | N | N |
| 10 | spring | 4 | restaurant | *M. domestica* | N | N | N | N | N | N | N | N | N | N | N | N | N |
| 11 | spring | 4 | restaurant | *M. domestica* | N | N | N | N | N | N | N | N | N | N | N | N | N |
| 12 | spring | 4 | restaurant | *M. domestica* | N | N | N | N | N | N | N | N | N | N | N | N | N |
| 13 | spring | 4 | restaurant | *M. domestica* | N | N | N | N | N | N | N | N | N | N | N | N | N |
| 14 | spring | 4 | parks | *L. sericata* | P | P | N | P | P | N | N | N | N | N | N | P | N |
| 15 | spring | 4 | residential areas | *L. sericata* | P | P | N | N | P | N | N | N | N | N | N | N | N |
| 16 | spring | 4 | farmers' markets | *L. sericata* | P | P | N | N | P | N | P | N | N | N | N | N | N |
| 17 | spring | 4 | farmers' markets | *L. sericata* | N | N | N | N | N | N | N | N | N | N | N | N | N |
| 18 | spring | 4 | parks | *B. peregrina* | N | N | N | N | N | N | N | N | N | N | N | N | N |
| 19 | spring | 4 | parks | *B. peregrina* | N | N | N | N | N | N | N | N | N | N | N | N | N |
| 20 | spring | 4 | residential areas | *B. peregrina* | P | P | N | N | P | N | N | N | N | N | N | N | N |
| 21 | spring | 4 | farmers' markets | *B. peregrina* | P | P | N | N | P | N | N | N | N | N | N | N | N |
| 22 | spring | 5 | residential areas | *M. domestica* | N | N | N | N | N | N | N | N | N | N | N | N | N |
| 23 | spring | 5 | residential areas | *M. domestica* | N | N | N | N | N | N | N | N | N | N | N | N | N |
| 24 | spring | 5 | residential areas | *M. domestica* | N | N | N | N | N | N | N | N | N | N | N | N | N |
| 25 | spring | 5 | residential areas | *M. domestica* | N | N | N | N | N | N | N | N | N | N | N | N | N |
| 26 | spring | 5 | farmers' markets | *M. domestica* | P | N | N | P | N | N | N | N | N | N | N | N | P |
| 27 | spring | 5 | farmers' markets | *M. domestica* | N | N | N | N | N | N | N | N | N | N | N | N | N |
| 28 | spring | 5 | farmers' markets | *M. domestica* | N | N | N | N | N | N | N | N | N | N | N | N | N |
| 29 | spring | 5 | restaurant | *M. domestica* | N | N | N | N | N | N | N | N | N | N | N | N | N |
| 30 | spring | 5 | restaurant | *M. domestica* | N | N | N | N | N | N | N | N | N | N | N | N | N |
| 31 | spring | 5 | restaurant | *M. domestica* | N | N | N | N | N | N | N | N | N | N | N | N | N |
| 32 | spring | 5 | restaurant | *M. domestica* | P | N | P | N | N | N | N | N | N | N | P | N | N |
| 33 | spring | 5 | restaurant | *M. domestica* | P | N | P | N | N | N | N | N | N | N | P | N | N |
| 34 | spring | 5 | parks | *L. sericata* | P | N | P | P | N | N | N | N | N | P | N | N | P |
| 35 | spring | 5 | residential areas | *L. sericata* | P | P | N | N | P | N | N | N | N | N | N | N | N |
| 36 | spring | 5 | farmers' markets | *L. sericata* | P | N | P | N | N | N | N | N | N | P | N | N | N |
| 37 | spring | 5 | parks | *B. peregrina* | P | P | P | N | P | N | N | N | N | P | N | N | N |
| 38 | spring | 5 | residential areas | *B. peregrina* | P | P | P | N | P | N | N | N | N | P | N | N | N |
| 39 | spring | 5 | farmers' markets | *B. peregrina* | P | P | N | N | P | N | N | N | N | N | N | N | N |
| 40 | summer | 6 | residential areas | *M. domestica* | P | P | N | N | P | N | N | N | N | N | N | N | N |
| 41 | summer | 6 | residential areas | *M. domestica* | P | P | N | P | P | N | N | N | N | N | N | P | N |
| 42 | summer | 6 | residential areas | *M. domestica* | P | N | P | N | N | N | N | N | N | N | P | N | N |
| 43 | summer | 6 | farmers' markets | *M. domestica* | N | N | N | N | N | N | N | N | N | N | N | N | N |
| 44 | summer | 6 | farmers' markets | *M. domestica* | P | N | P | N | N | N | N | N | N | N | P | N | N |
| 45 | summer | 6 | farmers' markets | *M. domestica* | P | N | N | P | N | N | N | N | N | N | N | P | N |
| 46 | summer | 6 | restaurant | *M. domestica* | N | N | N | N | N | N | N | N | N | N | N | N | N |
| 47 | summer | 6 | restaurant | *M. domestica* | N | N | N | N | N | N | N | N | N | N | N | N | N |
| 48 | summer | 6 | restaurant | *M. domestica* | P | P | P | N | P | N | N | N | N | N | P | N | N |
| 49 | summer | 6 | restaurant | *M. domestica* | N | N | N | N | N | N | N | N | N | N | N | N | N |
| 50 | summer | 6 | restaurant | *M. domestica* | P | P | N | N | P | N | N | N | N | N | N | N | N |
| 51 | summer | 6 | restaurant | *M. domestica* | N | N | N | N | N | N | N | N | N | N | N | N | N |
| 52 | summer | 6 | parks | *L. sericata* | P | P | P | P | P | N | N | N | N | P | N | N | P |
| 53 | summer | 6 | parks | *L. sericata* | P | P | N | P | P | N | N | N | N | N | N | N | P |
| 54 | summer | 6 | parks | *L. sericata* | P | P | P | P | P | N | N | N | N | P | N | N | P |
| 55 | summer | 6 | residential areas | *L. sericata* | P | P | P | P | P | N | P | N | N | P | P | N | P |
| 56 | summer | 6 | residential areas | *L. sericata* | P | P | N | P | P | N | P | N | N | N | N | N | P |
| 57 | summer | 6 | residential areas | *L. sericata* | P | P | N | N | P | N | N | N | N | N | N | N | N |
| 58 | summer | 6 | farmers' markets | *L. sericata* | N | N | N | N | N | N | N | N | N | N | N | N | N |
| 59 | summer | 6 | farmers' markets | *L. sericata* | P | P | P | P | P | N | N | N | N | P | N | N | P |
| 60 | summer | 6 | parks | *B. peregrina* | P | P | N | P | P | N | N | N | N | N | N | N | P |
| 61 | summer | 6 | residential areas | *B. peregrina* | P | P | P | N | P | N | N | N | N | N | P | N | N |
| 62 | summer | 6 | farmers' markets | *B. peregrina* | P | P | N | N | P | N | N | N | N | N | N | N | N |
| 63 | summer | 7 | residential areas | *M. domestica* | P | N | P | N | N | N | N | P | N | P | N | N | N |
| 64 | summer | 7 | residential areas | *M. domestica* | P | N | N | P | N | N | N | N | N | N | N | P | N |
| 65 | summer | 7 | residential areas | *M. domestica* | P | N | P | N | N | N | N | N | N | N | P | N | N |
| 66 | summer | 7 | residential areas | *M. domestica* | N | N | N | N | N | N | N | N | N | N | N | N | N |
| 67 | summer | 7 | residential areas | *M. domestica* | N | N | N | N | N | N | N | N | N | N | N | N | N |
| 68 | summer | 7 | residential areas | *M. domestica* | N | N | N | N | N | N | N | N | N | N | N | N | N |
| 69 | summer | 7 | farmers' markets | *M. domestica* | P | N | P | N | N | N | N | N | N | N | P | N | N |
| 70 | summer | 7 | farmers' markets | *M. domestica* | N | N | N | N | N | N | N | N | N | N | N | N | N |
| 71 | summer | 7 | farmers' markets | *M. domestica* | N | N | N | N | N | N | N | N | N | N | N | N | N |
| 72 | summer | 7 | farmers' markets | *M. domestica* | N | N | N | N | N | N | N | N | N | N | N | N | N |
| 73 | summer | 7 | farmers' markets | *M. domestica* | N | N | N | N | N | N | N | N | N | N | N | N | N |
| 74 | summer | 7 | restaurant | *M. domestica* | P | N | P | N | N | N | N | N | N | N | P | N | N |
| 75 | summer | 7 | restaurant | *M. domestica* | N | N | N | N | N | N | N | N | N | N | N | N | N |
| 76 | summer | 7 | restaurant | *M. domestica* | P | N | P | N | N | N | N | N | N | N | P | N | N |
| 77 | summer | 7 | restaurant | *M. domestica* | N | N | N | N | N | N | N | N | N | N | N | N | N |
| 78 | summer | 7 | restaurant | *M. domestica* | N | N | N | N | N | N | N | N | N | N | N | N | N |
| 79 | summer | 7 | restaurant | *M. domestica* | N | N | N | N | N | N | N | N | N | N | N | N | N |
| 80 | summer | 7 | parks | *L. sericata* | N | N | N | N | N | N | N | N | N | N | N | N | N |
| 81 | summer | 7 | parks | *L. sericata* | P | P | P | N | P | N | N | N | N | P | P | N | N |
| 82 | summer | 7 | residential areas | *L. sericata* | P | N | P | N | N | N | N | N | N | P | P | N | N |
| 83 | summer | 7 | residential areas | *L. sericata* | N | N | N | N | N | N | N | N | N | N | N | N | N |
| 84 | summer | 7 | residential areas | *L. sericata* | N | N | N | N | N | N | N | N | N | N | N | N | N |
| 85 | summer | 7 | residential areas | *L. sericata* | N | N | N | N | N | N | N | N | N | N | N | N | N |
| 86 | summer | 7 | farmers' markets | *L. sericata* | P | P | P | N | P | N | N | N | N | N | P | N | N |
| 87 | summer | 7 | parks | *B. peregrina* | P | P | P | N | P | N | N | N | N | N | P | N | N |
| 88 | summer | 7 | residential areas | *B. peregrina* | P | P | N | N | P | N | N | N | N | P | P | N | N |
| 89 | summer | 7 | residential areas | *B. peregrina* | N | N | N | N | N | N | N | N | N | N | N | N | N |
| 90 | summer | 7 | farmers' markets | *B. peregrina* | P | P | P | N | P | N | N | N | N | P | P | N | N |
| 91 | summer | 7 | farmers' markets | *B. peregrina* | N | N | N | N | N | N | N | N | N | N | N | N | N |
| 92 | summer | 7 | farmers' markets | *B. peregrina* | N | N | N | N | N | N | N | N | N | N | N | N | N |
| 93 | summer | 8 | residential areas | *M. domestica* | N | N | N | N | N | N | N | N | N | N | N | N | N |
| 94 | summer | 8 | residential areas | *M. domestica* | P | N | N | P | N | N | N | N | N | N | N | P | N |
| 95 | summer | 8 | residential areas | *M. domestica* | P | N | N | P | N | N | N | N | N | N | N | P | N |
| 96 | summer | 8 | residential areas | *M. domestica* | N | N | N | N | N | N | N | N | N | N | N | N | N |
| 97 | summer | 8 | residential areas | *M. domestica* | N | N | N | N | N | N | N | N | N | N | N | N | N |
| 98 | summer | 8 | residential areas | *M. domestica* | N | N | N | N | N | N | N | N | N | N | N | N | N |
| 99 | summer | 8 | farmers' markets | *M. domestica* | P | N | N | P | N | N | N | N | N | N | N | N | P |
| 100 | summer | 8 | farmers' markets | *M. domestica* | P | P | N | N | N | P | N | N | N | N | N | N | N |
| 101 | summer | 8 | farmers' markets | *M. domestica* | N | N | N | N | N | N | N | N | N | N | N | N | N |
| 102 | summer | 8 | farmers' markets | *M. domestica* | N | N | N | N | N | N | N | N | N | N | N | N | N |
| 103 | summer | 8 | restaurant | *M. domestica* | N | N | N | N | N | N | N | N | N | N | N | N | N |
| 104 | summer | 8 | restaurant | *M. domestica* | P | N | N | P | N | N | N | N | N | N | N | N | P |
| 105 | summer | 8 | restaurant | *M. domestica* | N | N | N | N | N | N | N | N | N | N | N | N | N |
| 106 | summer | 8 | restaurant | *M. domestica* | N | N | N | N | N | N | N | N | N | N | N | N | N |
| 107 | summer | 8 | parks | *L. sericata* | P | N | P | N | N | N | N | N | N | N | P | N | N |
| 108 | summer | 8 | parks | *L. sericata* | N | N | N | N | N | N | N | N | N | N | N | N | N |
| 109 | summer | 8 | residential areas | *L. sericata* | P | N | P | P | N | N | N | N | N | N | P | N | P |
| 110 | summer | 8 | residential areas | *L. sericata* | N | N | N | N | N | N | N | N | N | N | N | N | N |
| 111 | summer | 8 | farmers' markets | *L. sericata* | N | N | N | N | N | N | N | N | N | N | N | N | N |
| 112 | summer | 8 | farmers' markets | *L. sericata* | N | N | N | N | N | N | N | N | N | N | N | N | N |
| 113 | summer | 8 | parks | *B. peregrina* | N | N | N | N | N | N | N | N | N | N | N | N | N |
| 114 | summer | 8 | residential areas | *B. peregrina* | P | N | P | N | N | N | N | N | N | N | P | N | N |
| 115 | summer | 8 | residential areas | *B. peregrina* | N | N | N | N | N | N | N | N | N | N | N | N | N |
| 116 | summer | 8 | farmers' markets | *B. peregrina* | P | N | N | P | N | N | N | N | N | N | N | N | P |
| 117 | autumn | 9 | residential areas | *M. domestica* | N | N | N | N | N | N | N | N | N | N | N | N | N |
| 118 | autumn | 9 | residential areas | *M. domestica* | N | N | N | N | N | N | N | N | N | N | N | N | N |
| 119 | autumn | 9 | residential areas | *M. domestica* | N | N | N | N | N | N | N | N | N | N | N | N | N |
| 120 | autumn | 9 | residential areas | *M. domestica* | N | N | N | N | N | N | N | N | N | N | N | N | N |
| 121 | autumn | 9 | residential areas | *M. domestica* | N | N | N | N | N | N | N | N | N | N | N | N | N |
| 122 | autumn | 9 | farmers' markets | *M. domestica* | P | P | N | P | N | P | N | N | N | N | N | P | P |
| 123 | autumn | 9 | farmers' markets | *M. domestica* | P | N | P | P | N | N | N | N | N | N | P | P | N |
| 124 | autumn | 9 | farmers' markets | *M. domestica* | N | N | N | N | N | N | N | N | N | N | N | N | N |
| 125 | autumn | 9 | restaurant | *M. domestica* | N | N | N | N | N | N | N | N | N | N | N | N | N |
| 126 | autumn | 9 | restaurant | *M. domestica* | N | N | N | N | N | N | N | N | N | N | N | N | N |
| 127 | autumn | 9 | restaurant | *M. domestica* | N | N | N | N | N | N | N | N | N | N | N | N | N |
| 128 | autumn | 9 | restaurant | *M. domestica* | P | P | N | N | P | N | N | N | N | N | N | N | N |
| 129 | autumn | 9 | restaurant | *M. domestica* | P | P | P | N | P | N | N | N | N | N | P | N | N |
| 130 | autumn | 9 | restaurant | *M. domestica* | P | P | N | P | P | N | N | N | N | N | N | P | N |
| 131 | autumn | 9 | parks | *L. sericata* | P | N | P | N | N | N | N | N | P | N | P | N | N |
| 132 | autumn | 9 | residential areas | *L. sericata* | P | N | P | N | N | N | N | N | N | N | P | N | N |
| 133 | autumn | 9 | farmers' markets | *L. sericata* | P | N | P | P | N | N | N | N | P | N | P | P | N |
| 134 | autumn | 9 | parks | *B. peregrina* | P | N | P | N | N | N | N | N | N | N | P | N | N |
| 135 | autumn | 9 | residential areas | *B. peregrina* | P | N | P | N | N | N | N | N | N | N | P | N | N |
| 136 | autumn | 9 | farmers' markets | *B. peregrina* | P | P | P | N | P | N | N | P | N | N | P | N | N |
| 137 | autumn | 10 | residential areas | *M. domestica* | P | P | N | P | P | N | N | N | N | N | N | P | N |
| 138 | autumn | 10 | residential areas | *M. domestica* | P | N | N | P | N | N | N | N | N | N | N | P | N |
| 139 | autumn | 10 | residential areas | *M. domestica* | P | N | N | P | N | N | N | N | N | N | N | P | N |
| 140 | autumn | 10 | residential areas | *M. domestica* | N | N | N | N | N | N | N | N | N | N | N | N | N |
| 141 | autumn | 10 | residential areas | *M. domestica* | N | N | N | N | N | N | N | N | N | N | N | N | N |
| 142 | autumn | 10 | farmers' markets | *M. domestica* | P | N | N | P | N | N | N | N | N | N | N | P | P |
| 143 | autumn | 10 | farmers' markets | *M. domestica* | P | N | N | P | N | N | N | N | N | N | N | P | N |
| 144 | autumn | 10 | farmers' markets | *M. domestica* | N | N | N | N | N | N | N | N | N | N | N | N | N |
| 145 | autumn | 10 | farmers' markets | *M. domestica* | N | N | N | N | N | N | N | N | N | N | N | N | N |
| 146 | autumn | 10 | farmers' markets | *M. domestica* | N | N | N | N | N | N | N | N | N | N | N | N | N |
| 147 | autumn | 10 | restaurant | *M. domestica* | P | N | N | P | N | N | N | N | N | N | N | P | N |
| 148 | autumn | 10 | restaurant | *M. domestica* | P | N | N | P | N | N | N | N | N | N | N | P | N |
| 149 | autumn | 10 | restaurant | *M. domestica* | P | N | N | P | N | N | N | N | N | N | N | P | N |
| 150 | autumn | 10 | restaurant | *M. domestica* | P | N | N | P | N | N | N | N | N | N | N | P | N |
| 151 | autumn | 10 | restaurant | *M. domestica* | N | N | N | N | N | N | N | N | N | N | N | N | N |
| 152 | autumn | 10 | restaurant | *M. domestica* | N | N | N | N | N | N | N | N | N | N | N | N | N |
| 153 | autumn | 10 | restaurant | *M. domestica* | N | N | N | N | N | N | N | N | N | N | N | N | N |
| 154 | autumn | 10 | parks | *L. sericata* | P | P | N | N | P | N | N | N | N | N | N | N | N |
| 155 | autumn | 10 | residential areas | *L. sericata* | P | P | P | N | P | N | N | N | N | P | N | N | N |
| 156 | autumn | 10 | farmers' markets | *L. sericata* | P | P | N | N | P | N | N | N | N | N | N | N | N |
| 157 | autumn | 10 | farmers' markets | *L. sericata* | N | N | N | N | N | N | N | N | N | N | N | N | N |
| 158 | autumn | 10 | parks | *B. peregrina* | P | P | P | N | P | N | N | N | N | P | N | N | N |
| 159 | autumn | 10 | residential areas | *B. peregrina* | P | P | P | N | P | N | N | N | N | P | N | N | N |
| 160 | autumn | 10 | farmers' markets | *B. peregrina* | P | P | P | P | P | N | N | N | N | P | N | P | N |
| 161 | autumn | 11 | residential areas | *M. domestica* | P | N | N | P | N | N | N | N | N | N | N | P | P |
| 162 | autumn | 11 | residential areas | *M. domestica* | P | N | N | P | N | N | N | N | N | N | N | P | N |
| 163 | autumn | 11 | residential areas | *M. domestica* | N | N | N | N | N | N | N | N | N | N | N | N | N |
| 164 | autumn | 11 | residential areas | *M. domestica* | N | N | N | N | N | N | N | N | N | N | N | N | N |
| 165 | autumn | 11 | residential areas | *M. domestica* | N | N | N | N | N | N | N | N | N | N | N | N | N |
| 166 | autumn | 11 | residential areas | *M. domestica* | N | N | N | N | N | N | N | N | N | N | N | N | N |
| 167 | autumn | 11 | farmers' markets | *M. domestica* | P | N | N | P | N | N | N | N | N | N | N | P | N |
| 168 | autumn | 11 | farmers' markets | *M. domestica* | N | N | N | N | N | N | N | N | N | N | N | N | N |
| 169 | autumn | 11 | farmers' markets | *M. domestica* | N | N | N | N | N | N | N | N | N | N | N | N | N |
| 170 | autumn | 11 | farmers' markets | *M. domestica* | N | N | N | N | N | N | N | N | N | N | N | N | N |
| 171 | autumn | 11 | farmers' markets | *M. domestica* | N | N | N | N | N | N | N | N | N | N | N | N | N |
| 172 | autumn | 11 | farmers' markets | *M. domestica* | N | N | N | N | N | N | N | N | N | N | N | N | N |
| 173 | autumn | 11 | restaurant | *M. domestica* | P | N | N | P | N | N | N | N | N | N | N | P | N |
| 174 | autumn | 11 | restaurant | *M. domestica* | P | N | N | P | N | N | N | N | N | N | N | P | N |
| 175 | autumn | 11 | restaurant | *M. domestica* | P | N | N | P | N | N | N | N | N | N | N | P | N |
| 176 | autumn | 11 | restaurant | *M. domestica* | N | N | N | N | N | N | N | N | N | N | N | N | N |
| 177 | autumn | 11 | restaurant | *M. domestica* | N | N | N | N | N | N | N | N | N | N | N | N | N |
| 178 | autumn | 11 | restaurant | *M. domestica* | N | N | N | N | N | N | N | N | N | N | N | N | N |
| 179 | autumn | 11 | restaurant | *M. domestica* | N | N | N | N | N | N | N | N | N | N | N | N | N |
| 180 | autumn | 11 | parks | *L. sericata* | P | P | N | P | P | N | N | N | N | N | N | P | N |
| 181 | autumn | 11 | residential areas | *L. sericata* | P | P | P | P | P | N | N | N | N | P | N | P | N |
| 182 | autumn | 11 | residential areas | *L. sericata* | N | N | N | N | N | N | N | N | N | N | N | N | N |
| 183 | autumn | 11 | farmers' markets | *L. sericata* | P | P | P | N | P | N | N | N | N | P | N | N | N |
| 184 | autumn | 11 | farmers' markets | *L. sericata* | N | N | N | N | N | N | N | N | N | N | N | N | N |
| 185 | autumn | 11 | parks | *B. peregrina* | P | P | N | N | P | N | N | N | N | N | N | N | N |
| 186 | autumn | 11 | parks | *B. peregrina* | N | N | N | N | N | N | N | N | N | N | N | N | N |
| 187 | autumn | 11 | residential areas | *B. peregrina* | P | N | N | P | N | N | N | N | N | N | N | P | N |
| 188 | autumn | 11 | residential areas | *B. peregrina* | N | N | N | N | N | N | N | N | N | N | N | N | N |
| 189 | autumn | 11 | farmers' markets | *B. peregrina* | P | P | P | N | P | N | N | N | N | P | N | N | N |

N means Negative；P means Positive
