# Supplementary material for: Unveiling the hidden dangers: enteropathogens carried by flies in Pudong New Area
Source: BMC Infect Dis. 2024 Jun 7;24:569. doi: 10.1186/s12879-024-09448-0 (PMC11162034; doi:10.1186/s12879-024-09448-0)
Supplement: Supplementary file 2 — Supplementary Material 2 [file 12879_2024_9448_MOESM2_ESM.docx]

| **Enteropathogens harbored by human beings** | | | | | | | | | | | | | | | | |
| --- | --- | --- | --- | --- | --- | --- | --- | --- | --- | --- | --- | --- | --- | --- | --- | --- |
| **year** | **month** | **sample** | **positive** | **rate** | ***E. coli*** | ***V. parahaemolyticus*** | **Salmonella** | ***Aeromonas hydrophila*** | **Plesiomonas shigelloides** | ***Yersinia enterocolitica*** | **Shigella** | **Rotavirus** | **Norovirus** | **Adenovirus** | **Astrovirus** | **Sapovirus** |
| 2021 | 4 | 42 | 15 | 35.71 | 3 | 0 | 2 | 0 | 0 | 0 | 0 | 0 | 8 | 2 | 0 | 1 |
|  | 5 | 48 | 19 | 39.58 | 3 | 0 | 2 | 0 | 2 | 0 | 0 | 2 | 9 | 1 | 2 | 0 |
|  | 6 | 61 | 18 | 29.51 | 7 | 1 | 5 | 0 | 0 | 0 | 0 | 1 | 1 | 1 | 4 | 2 |
|  | 7 | 84 | 42 | 50.00 | 12 | 2 | 6 | 0 | 0 | 1 | 0 | 1 | 5 | 6 | 11 | 1 |
|  | 8 | 58 | 30 | 51.72 | 12 | 2 | 14 | 0 | 0 | 0 | 0 | 0 | 0 | 3 | 4 | 0 |
|  | 9 | 34 | 13 | 38.24 | 5 | 5 | 1 | 0 | 0 | 0 | 0 | 3 | 0 | 0 | 0 | 0 |
|  | 10 | 23 | 10 | 43.48 | 4 | 1 | 1 | 0 | 0 | 0 | 0 | 2 | 5 | 0 | 0 | 0 |
|  | 11 | 26 | 11 | 42.31 | 2 | 0 | 0 | 0 | 0 | 0 | 0 | 2 | 6 | 2 | 0 | 1 |
