# Supplementary material for: Unveiling the hidden dangers: enteropathogens carried by flies in Pudong New Area
Source: BMC Infect Dis. 2024 Jun 7;24:569. doi: 10.1186/s12879-024-09448-0 (PMC11162034; doi:10.1186/s12879-024-09448-0)
Supplement: Supplementary file 3 — Supplementary Material 3 [file 12879_2024_9448_MOESM3_ESM.docx]

|  | | | |  |  | | **Fly collection information** | | | | | | | | | | | |  |
| --- | --- | --- | --- | --- | --- | --- | --- | --- | --- | --- | --- | --- | --- | --- | --- | --- | --- | --- | --- |
| ID | Month | Community | **Cages** | **Collection point** | **North latitude** | **East longitude** | | **Habitat** | ***M. domestica*** | ***L. sericata*** | ***B. peregrina*** | **Other species of Sarcophaga** | **Other species of Lucilia** | ***Chrysomyia megacephala*** | ***Muscina stabulans*** | ***Musca sorbens*** | ***Fannia canicularis*** | ***Fannia prisca*** | |
| 1 | 4 | Gaoqiao | 20 | Waigaoqiao Nijin 3D Plaza (Zhangjia Street) | 31°20′45.01″ | 121°34′48.01″ | | residential areas | 4 | 2 | 2 | 0 | 0 | 0 | 0 | 0 | 0 | 0 | |
| 2 | 4 | Gaoqiao | 20 | Gaoqiao Vegetable Market (No. 2 Wangjia Street) | 31°20′53.81″ | 121°34′51.10″ | | farmers' markets | 3 | 3 | 1 | 0 | 0 | 0 | 0 | 0 | 0 | 0 | |
| 3 | 4 | Gaoqiao | 20 | Collection of Books Lamb Restaurant (Huashan Road Branch) (1F, No. 804 Huashan Road) | 31°20′55.77″ | 121°34′45.34″ | | restaurants | 8 | 0 | 0 | 1 | 0 | 0 | 0 | 0 | 0 | 0 | |
| 4 | 4 | Gaoqiao | 20 | Gaoqiao Park (No. 482 Huashan Road) | 31°20′48.41″ | 121°34′21.61″ | | parks | 0 | 2 | 4 | 1 | 0 | 0 | 0 | 0 | 0 | 0 | |
| 5 | 4 | Gaohang | 20 | Longju Apartment | 31°15′18.97″ | 121°33′39.07″ | | residential areas | 5 | 3 | 1 | 0 | 0 | 0 | 0 | 0 | 0 | 0 | |
| 6 | 4 | Gaohang | 20 | Huagao Farmers' Market (No. 1073 Jin Gao Road) | 31°16′55.24″ | 121°36′36.97″ | | farmers' markets | 6 | 2 | 4 | 1 | 0 | 0 | 0 | 0 | 0 | 0 | |
| 7 | 4 | Gaohang | 20 | Jin Gao Restaurant (No. 217 Jin Gao Road) | 31°17′44.59″ | 121°36′26.17″ | | restaurants | 5 | 0 | 0 | 0 | 0 | 0 | 0 | 0 | 0 | 0 | |
| 8 | 4 | Gaohang | 20 | Gaohang Green Park (No. 906 Jinjing Road) | 31°17′14.36″ | 121°36′53.21″ | | parks | 0 | 1 | 0 | 0 | 0 | 0 | 0 | 0 | 0 | 0 | |
| 9 | 4 | Nanhui | 20 | Luchaogang 7th Group (Jiangshan Road) | 30°52′12.38″ | 121°51′34.35″ | | residential areas | 4 | 2 | 3 | 1 | 0 | 0 | 0 | 0 | 0 | 0 | |
| 10 | 4 | Nanhui | 20 | Luchaogang Seafood Market (No. 618 Yugang Road) | 30°51′31.32″ | 121°51′2.52″ | | farmers' markets | 3 | 2 | 2 | 1 | 0 | 0 | 0 | 0 | 0 | 0 | |
| 11 | 4 | Nanhui | 20 | Lingang Zero Point Barbecue City (No. 138, Lane 1889, Jiangshan Road) | 30°52′11.57″ | 121°50′50.52″ | | restaurants | 3 | 0 | 0 | 0 | 0 | 0 | 0 | 0 | 0 | 0 | |
| 12 | 4 | Nanhui | 20 | Luchaogang Park in Nanhui New Town (230 meters east of the intersection of Chaohe Road and Jiangshan Road in Nanhui New Town) | 30°52′8.07″ | 121°51′46.51″ | | parks | 0 | 0 | 4 | 1 | 0 | 0 | 0 | 0 | 0 | 0 | |
| 13 | 4 | Zhuqiao | 20 | Huaxing Village 6 (about 100 meters southwest of Shunchuan Apartment, Xiazhou Road) | 31°12′2.78″ | 121°42′56.64″ | | residential areas | 3 | 1 | 2 | 0 | 0 | 0 | 0 | 0 | 0 | 0 | |
| 14 | 4 | Zhuqiao | 20 | Zhuqiao Trade Market (Hui Shang Apartment Branch) (No. 38 Chuangxin Street) | 31°06′57.51″ | 121°45′33.74″ | | farmers' markets | 5 | 2 | 4 | 0 | 1 | 0 | 0 | 0 | 0 | 0 | |
| 15 | 4 | Zhuqiao | 20 | Tianhe Plaza (No. 666 Baixi Road) | 31°06′59.58″ | 121°45′39.55″ | | restaurants | 10 | 0 | 0 | 1 | 1 | 0 | 0 | 0 | 0 | 0 | |
| 16 | 4 | Zhuqiao | 20 | Haitianhu Park (about 50 meters southeast of the intersection of Weiting Road and Baixi Road (about 50 meters west of Zhuqiao Town Cultural Center)) | 31°06′4.07″ | 121°45′50.62″ | | parks | 0 | 3 | 0 | 1 | 0 | 0 | 0 | 0 | 0 | 0 | |
| 17 | 4 | Sanlin | 20 | Residential Community at No. 334 Lingzhao Road (No. 26, Lane 334 Lingzhao Road) | 31°08′32.17″ | 121°29′49.84″ | | residential areas | 4 | 2 | 2 | 1 | 1 | 0 | 0 | 0 | 0 | 0 | |
| 18 | 4 | Sanlin | 20 | Lingyang Farmers' Market (Room Z-1, 1F, No. 3787 Shangnan Road, H Area, No. 13) | 31°09′6.34″ | 121°30′18.11″ | | farmers' markets | 6 | 3 | 3 | 2 | 2 | 0 | 0 | 0 | 0 | 0 | |
| 19 | 4 | Sanlin | 20 | Legendary Clay Pot (Changqing Road Branch) (No. 2068 Changqing Road) | 31°08′22.31″ | 121°29′49.88″ | | restaurants | 4 | 0 | 0 | 1 | 1 | 0 | 0 | 0 | 0 | 0 | |
| 20 | 4 | Sanlin | 20 | Sanlin Riverside Green Space (140 meters northwest of the intersection of Yanjiang Road and S20 Outer Ring Expressway Auxiliary Road) | 31°07′50.73″ | 121°28′24.94″ | | parks | 0 | 4 | 0 | 1 | 0 | 0 | 0 | 0 | 0 | 0 | |
| 21 | 4 | Tanqiao | 20 | No. 32 Weishan 2nd Village | 31°12′29.25″ | 121°30′48.92″ | | residential areas | 4 | 3 | 3 | 0 | 0 | 0 | 0 | 0 | 0 | 0 | |
| 22 | 4 | Tanqiao | 20 | Pushang Vegetable Market (Nanquan Branch) (No. 1315 Nanquan Road, near Tangqiao Road) | 31°12′36.14″ | 121°31′19.52″ | | farmers' markets | 4 | 1 | 2 | 0 | 0 | 0 | 0 | 0 | 0 | 0 | |
| 23 | 4 | Tanqiao | 20 | Haidilao Hotpot (Paris Spring Pujian Branch) (No. 118 Pujian Road) | 31°12′30.67″ | 121°31′15.17″ | | restaurants | 7 | 0 | 0 | 1 | 0 | 0 | 0 | 0 | 0 | 0 | |
| 24 | 4 | Tanqiao | 20 | Nanpu Square Park (No. 2277 Pudong South Road) | 31°12′13.97″ | 121°30′51.52″ | | parks | 0 | 1 | 0 | 5 | 0 | 0 | 0 | 0 | 0 | 0 | |
| 25 | 4 | Nicheng | 20 | Qingfeng Liyuan (No. 779 Yunhan Road) | 30°54′33.30″ | 121°48′38.59″ | | residential areas | 1 | 1 | 2 | 0 | 1 | 0 | 0 | 0 | 0 | 0 | |
| 26 | 4 | Nicheng | 20 | Fajia Fruit Wholesale (Qiuyu Road Branch) (No. 726 Qiuyu Road) | 30°54′30.85″ | 121°48′58.54″ | | farmers' markets | 2 | 3 | 3 | 1 | 1 | 0 | 0 | 0 | 0 | 0 | |
| 27 | 4 | Nicheng | 20 | Lingang Baolong Plaza (No. 128, Lane 3155 Hongyin Road) | 30°54′22.35″ | 121°48′56.97″ | | restaurants | 13 | 0 | 0 | 2 | 1 | 0 | 0 | 0 | 0 | 0 | |
| 28 | 4 | Nicheng | 20 | Nicheng Riverside Cultural Park (intersection of Pengping Road and Chiyue Road) | 30°54′18.38″ | 121°48′33.39″ | | parks | 0 | 2 | 3 | 1 | 0 | 0 | 0 | 0 | 0 | 0 | |
| 29 | 4 | Huinan | 20 | Dangwan New Village (No. 2 Dangwan Road) | 31°02′39.34″ | 121°45′53.55″ | | residential areas | 4 | 1 | 3 | 0 | 0 | 0 | 0 | 0 | 0 | 0 | |
| 30 | 4 | Huinan | 20 | Dangwan Vegetable Market (Jinghai Road Nanhui Flower and Bird Market) | 31°02′42.04″ | 121°46′3.28″ | | farmers' markets | 0 | 1 | 0 | 2 | 1 | 0 | 0 | 0 | 0 | 0 | |
| 31 | 4 | Huinan | 20 | Guizhou Yellow Beef Hotpot (No. 126 Jinghai Road) | 31°02′40.81″ | 121°46′6.74″ | | restaurants | 8 | 0 | 0 | 0 | 0 | 0 | 0 | 0 | 0 | 0 | |
| 32 | 4 | Huinan | 20 | Nanhui Peach Blossom Village (No. 289 Beimen Road) | 31°03′41.18″ | 121°45′20.02″ | | parks | 0 | 4 | 5 | 1 | 0 | 0 | 0 | 0 | 0 | 0 | |
| 33 | 4 | Caolu | 20 | Jinhai Huacheng Yunxiu Jiayuan (Lane 57, Xiayu Road) | 31°15′21.74″ | 121°41′9.58″ | | residential areas | 5 | 2 | 1 | 0 | 1 | 0 | 0 | 0 | 0 | 0 | |
| 34 | 4 | Caolu | 20 | Yuexing Farmers' Market (Gonglu Vegetable Market Branch) (Room 101-1B, No. 48, Lane 235 Gonghua Road, No. 14-15) | 31°15′53.35″ | 121°41′8.52″ | | farmers' markets | 1 | 2 | 0 | 0 | 2 | 0 | 0 | 0 | 0 | 0 | |
| 35 | 4 | Caolu | 20 | Henan Mixed Noodles and Braised Beef Noodles at Gonglu Xincheng (No. 488 Gonghua Road, Gonghua New Village) | 31°15′33.12″ | 121°41′13.38″ | | restaurants | 11 | 0 | 0 | 0 | 2 | 0 | 0 | 0 | 0 | 0 | |
| 36 | 4 | Caolu | 20 | Firefighting Street Corner Theme Park (northwest of the intersection of Jinhai Road and Lingkong Road) | 31°16′19.67″ | 121°41′12.99″ | | parks | 0 | 2 | 1 | 1 | 0 | 0 | 0 | 0 | 0 | 0 | |
| 37 | 4 | Chuansha | 20 | Xiangchuan Homeland (No. 1-74, Lane 458 Xiangchuan Road) | 31°10′58.12″ | 121°41′42.40″ | | residential areas | 6 | 3 | 1 | 2 | 1 | 0 | 0 | 0 | 0 | 0 | |
| 38 | 4 | Chuansha | 20 | Chuansha Farmers' Market (No. 401 Chuanhuan South Road, Chuanhuang Road) | 31°11′16.80″ | 121°42′19.42″ | | farmers' markets | 7 | 5 | 1 | 1 | 0 | 0 | 0 | 0 | 0 | 0 | |
| 39 | 4 | Chuansha | 20 | Chuansha Commercial Plaza (No. 2, Lane 1136 Miaojing Road, Chuansha New Town) | 31°10′49.97″ | 121°41′35.54″ | | restaurants | 7 | 0 | 0 | 0 | 1 | 0 | 0 | 0 | 0 | 0 | |
| 40 | 4 | Chuansha | 20 | Chuansha Park (No. 437 Chengnan Road) | 31°11′27.06″ | 121°42′3.34″ | | parks | 0 | 1 | 4 | 1 | 0 | 1 | 0 | 0 | 0 | 0 | |
| 41 | 5 | Gaoqiao | 20 | Waigaoqiao Nijin 3D Plaza (Zhangjia Street) | 31°20′45.01″ | 121°34′48.01″ | | residential areas | 2 | 0 | 0 | 0 | 1 | 1 | 0 | 0 | 0 | 0 | |
| 42 | 5 | Gaoqiao | 20 | Gaoqiao Vegetable Market (No. 2 Wangjia Street) | 31°20′53.81″ | 121°34′51.10″ | | farmers' markets | 0 | 3 | 0 | 1 | 0 | 0 | 0 | 0 | 0 | 0 | |
| 43 | 5 | Gaoqiao | 20 | Collection of Books Lamb Restaurant (Huashan Road Branch) (1F, No. 804 Huashan Road) | 31°20′55.77″ | 121°34′45.34″ | | restaurants | 8 | 0 | 0 | 0 | 1 | 1 | 0 | 0 | 0 | 0 | |
| 44 | 5 | Gaoqiao | 20 | Gaoqiao Park (No. 482 Huashan Road) | 31°20′48.41″ | 121°34′21.61″ | | parks | 1 | 3 | 0 | 2 | 0 | 0 | 0 | 0 | 0 | 0 | |
| 45 | 5 | Gaohang | 20 | Longju Apartment | 31°15′18.97″ | 121°33′39.07″ | | residential areas | 5 | 3 | 2 | 1 | 1 | 0 | 1 | 0 | 0 | 0 | |
| 46 | 5 | Gaohang | 20 | Huagao Farmers' Market (No. 1073 Jin Gao Road) | 31°16′55.24″ | 121°36′36.97″ | | farmers' markets | 4 | 4 | 1 | 1 | 0 | 1 | 0 | 0 | 0 | 0 | |
| 47 | 5 | Gaohang | 20 | Jin Gao Restaurant (No. 217 Jin Gao Road) | 31°17′44.59″ | 121°36′26.17″ | | restaurants | 4 | 0 | 1 | 1 | 2 | 1 | 1 | 0 | 0 | 0 | |
| 48 | 5 | Gaohang | 20 | Gaohang Green Park (No. 906 Jinjing Road) | 31°17′14.36″ | 121°36′53.21″ | | parks | 0 | 0 | 2 | 1 | 0 | 1 | 0 | 0 | 0 | 0 | |
| 49 | 5 | Nanhui | 20 | Luchaogang 7th Group (Jiangshan Road) | 30°52′12.38″ | 121°51′34.35″ | | residential areas | 6 | 2 | 1 | 2 | 1 | 2 | 1 | 1 | 0 | 0 | |
| 50 | 5 | Nanhui | 20 | Luchaogang Seafood Market (No. 618 Yugang Road) | 30°51′31.32″ | 121°51′2.52″ | | farmers' markets | 1 | 2 | 2 | 2 | 2 | 0 | 0 | 0 | 0 | 0 | |
| 51 | 5 | Nanhui | 20 | Lingang Zero Point Barbecue City (No. 138, Lane 1889, Jiangshan Road) | 30°52′11.57″ | 121°50′50.52″ | | restaurants | 5 | 0 | 0 | 1 | 1 | 1 | 0 | 0 | 0 | 0 | |
| 52 | 5 | Nanhui | 20 | Luchaogang Park in Nanhui New Town (230 meters east of the intersection of Chaohe Road and Jiangshan Road in Nanhui New Town) | 30°52′8.07″ | 121°51′46.51″ | | parks | 0 | 3 | 0 | 0 | 0 | 0 | 0 | 0 | 0 | 0 | |
| 53 | 5 | Zhuqiao | 20 | Huaxing Village 6 (about 100 meters southwest of Shunchuan Apartment, Xiazhou Road) | 31°12′2.78″ | 121°42′56.64″ | | residential areas | 7 | 3 | 2 | 0 | 1 | 0 | 0 | 0 | 0 | 0 | |
| 54 | 5 | Zhuqiao | 20 | Zhuqiao Trade Market (Hui Shang Apartment Branch) (No. 38 Chuangxin Street) | 31°06′57.51″ | 121°45′33.74″ | | farmers' markets | 1 | 1 | 3 | 2 | 1 | 0 | 0 | 0 | 0 | 0 | |
| 55 | 5 | Zhuqiao | 20 | Tianhe Plaza (No. 666 Baixi Road) | 31°06′59.58″ | 121°45′39.55″ | | restaurants | 4 | 0 | 0 | 2 | 1 | 1 | 0 | 0 | 0 | 0 | |
| 56 | 5 | Zhuqiao | 20 | Haitianhu Park (about 50 meters southeast of the intersection of Weiting Road and Baixi Road (about 50 meters west of Zhuqiao Town Cultural Center)) | 31°06′4.07″ | 121°45′50.62″ | | parks | 1 | 4 | 1 | 1 | 0 | 0 | 0 | 1 | 0 | 0 | |
| 57 | 5 | Sanlin | 20 | Residential Community at No. 334 Lingzhao Road (No. 26, Lane 334 Lingzhao Road) | 31°08′32.17″ | 121°29′49.84″ | | residential areas | 5 | 1 | 4 | 0 | 0 | 1 | 0 | 0 | 0 | 0 | |
| 58 | 5 | Sanlin | 20 | Lingyang Farmers' Market (Room Z-1, 1F, No. 3787 Shangnan Road, H Area, No. 13) | 31°09′6.34″ | 121°30′18.11″ | | farmers' markets | 5 | 0 | 1 | 1 | 1 | 1 | 1 | 0 | 0 | 0 | |
| 59 | 5 | Sanlin | 20 | Legendary Clay Pot (Changqing Road Branch) (No. 2068 Changqing Road) | 31°08′22.31″ | 121°29′49.88″ | | restaurants | 6 | 1 | 0 | 0 | 0 | 1 | 0 | 0 | 0 | 0 | |
| 60 | 5 | Sanlin | 20 | Sanlin Riverside Green Space (140 meters northwest of the intersection of Yanjiang Road and S20 Outer Ring Expressway Auxiliary Road) | 31°07′50.73″ | 121°28′24.94″ | | parks | 0 | 2 | 2 | 0 | 1 | 0 | 0 | 0 | 0 | 0 | |
| 61 | 5 | Tanqiao | 20 | No. 32 Weishan 2nd Village | 31°12′29.25″ | 121°30′48.92″ | | residential areas | 6 | 2 | 4 | 3 | 2 | 2 | 0 | 0 | 0 | 0 | |
| 62 | 5 | Tanqiao | 20 | Pushang Vegetable Market (Nanquan Branch) (No. 1315 Nanquan Road, near Tangqiao Road) | 31°12′36.14″ | 121°31′19.52″ | | farmers' markets | 0 | 2 | 3 | 2 | 0 | 1 | 0 | 0 | 0 | 0 | |
| 63 | 5 | Tanqiao | 20 | Haidilao Hotpot (Paris Spring Pujian Branch) (No. 118 Pujian Road) | 31°12′30.67″ | 121°31′15.17″ | | restaurants | 6 | 0 | 0 | 0 | 2 | 1 | 0 | 0 | 0 | 0 | |
| 64 | 5 | Tanqiao | 20 | Nanpu Square Park (No. 2277 Pudong South Road) | 31°12′13.97″ | 121°30′51.52″ | | parks | 1 | 1 | 3 | 2 | 0 | 0 | 0 | 0 | 0 | 0 | |
| 65 | 5 | Nicheng | 20 | Qingfeng Liyuan (No. 779 Yunhan Road) | 30°54′33.30″ | 121°48′38.59″ | | residential areas | 5 | 3 | 2 | 1 | 1 | 1 | 0 | 0 | 0 | 0 | |
| 66 | 5 | Nicheng | 20 | Fajia Fruit Wholesale (Qiuyu Road Branch) (No. 726 Qiuyu Road) | 30°54′30.85″ | 121°48′58.54″ | | farmers' markets | 6 | 3 | 1 | 0 | 1 | 2 | 0 | 0 | 0 | 0 | |
| 67 | 5 | Nicheng | 20 | Lingang Baolong Plaza (No. 128, Lane 3155 Hongyin Road) | 30°54′22.35″ | 121°48′56.97″ | | restaurants | 6 | 0 | 0 | 2 | 2 | 1 | 0 | 0 | 0 | 0 | |
| 68 | 5 | Nicheng | 20 | Nicheng Riverside Cultural Park (intersection of Pengping Road and Chiyue Road) | 30°54′18.38″ | 121°48′33.39″ | | parks | 0 | 2 | 5 | 1 | 0 | 0 | 0 | 0 | 0 | 0 | |
| 69 | 5 | Huinan | 20 | Dangwan New Village (No. 2 Dangwan Road) | 31°02′39.34″ | 121°45′53.55″ | | residential areas | 4 | 1 | 1 | 0 | 2 | 1 | 0 | 0 | 0 | 0 | |
| 70 | 5 | Huinan | 20 | Dangwan Vegetable Market (Jinghai Road Nanhui Flower and Bird Market) | 31°02′42.04″ | 121°46′3.28″ | | farmers' markets | 2 | 2 | 1 | 0 | 1 | 1 | 0 | 0 | 0 | 0 | |
| 71 | 5 | Huinan | 20 | Guizhou Yellow Beef Hotpot (No. 126 Jinghai Road) | 31°02′40.81″ | 121°46′6.74″ | | restaurants | 1 | 0 | 0 | 2 | 1 | 1 | 0 | 0 | 0 | 0 | |
| 72 | 5 | Huinan | 20 | Nanhui Peach Blossom Village (No. 289 Beimen Road) | 31°03′41.18″ | 121°45′20.02″ | | parks | 1 | 0 | 3 | 0 | 0 | 0 | 1 | 0 | 0 | 0 | |
| 73 | 5 | Caolu | 20 | Jinhai Huacheng Yunxiu Jiayuan (Lane 57, Xiayu Road) | 31°15′21.74″ | 121°41′9.58″ | | residential areas | 5 | 2 | 2 | 2 | 0 | 1 | 1 | 0 | 0 | 0 | |
| 74 | 5 | Caolu | 20 | Yuexing Farmers' Market (Gonglu Vegetable Market Branch) (Room 101-1B, No. 48, Lane 235 Gonghua Road, No. 14-15) | 31°15′53.35″ | 121°41′8.52″ | | farmers' markets | 1 | 1 | 2 | 5 | 1 | 2 | 1 | 0 | 0 | 0 | |
| 75 | 5 | Caolu | 20 | Henan Mixed Noodles and Braised Beef Noodles at Gonglu Xincheng (No. 488 Gonghua Road, Gonghua New Village) | 31°15′33.12″ | 121°41′13.38″ | | restaurants | 6 | 0 | 1 | 0 | 1 | 2 | 1 | 0 | 0 | 0 | |
| 76 | 5 | Caolu | 20 | Firefighting Street Corner Theme Park (northwest of the intersection of Jinhai Road and Lingkong Road) | 31°16′19.67″ | 121°41′12.99″ | | parks | 0 | 5 | 1 | 1 | 0 | 1 | 0 | 0 | 0 | 0 | |
| 77 | 5 | Chuansha | 20 | Xiangchuan Homeland (No. 1-74, Lane 458 Xiangchuan Road) | 31°10′58.12″ | 121°41′42.40″ | | residential areas | 9 | 3 | 2 | 0 | 0 | 1 | 1 | 1 | 0 | 0 | |
| 78 | 5 | Chuansha | 20 | Chuansha Farmers' Market (No. 401 Chuanhuan South Road, Chuanhuang Road) | 31°11′16.80″ | 121°42′19.42″ | | farmers' markets | 2 | 1 | 4 | 2 | 2 | 1 | 1 | 0 | 0 | 0 | |
| 79 | 5 | Chuansha | 20 | Chuansha Commercial Plaza (No. 2, Lane 1136 Miaojing Road, Chuansha New Town) | 31°10′49.97″ | 121°41′35.54″ | | restaurants | 5 | 0 | 0 | 2 | 1 | 1 | 0 | 0 | 0 | 0 | |
| 80 | 5 | Chuansha | 20 | Chuansha Park (No. 437 Chengnan Road) | 31°11′27.06″ | 121°42′3.34″ | | parks | 0 | 0 | 3 | 1 | 0 | 2 | 0 | 0 | 0 | 0 | |
| 81 | 6 | Gaoqiao | 20 | Waigaoqiao Nijin 3D Plaza (Zhangjia Street) | 31°20′45.01″ | 121°34′48.01″ | | residential areas | 6 | 4 | 0 | 0 | 0 | 2 | 1 | 1 | 0 | 0 | |
| 82 | 6 | Gaoqiao | 20 | Gaoqiao Vegetable Market (No. 2 Wangjia Street) | 31°20′53.81″ | 121°34′51.10″ | | farmers' markets | 4 | 3 | 2 | 0 | 2 | 0 | 0 | 0 | 0 | 0 | |
| 83 | 6 | Gaoqiao | 20 | Collection of Books Lamb Restaurant (Huashan Road Branch) (1F, No. 804 Huashan Road) | 31°20′55.77″ | 121°34′45.34″ | | restaurants | 8 | 0 | 0 | 0 | 2 | 2 | 1 | 0 | 0 | 1 | |
| 84 | 6 | Gaoqiao | 20 | Gaoqiao Park (No. 482 Huashan Road) | 31°20′48.41″ | 121°34′21.61″ | | parks | 1 | 4 | 1 | 4 | 0 | 0 | 1 | 2 | 0 | 1 | |
| 85 | 6 | Gaohang | 20 | Longju Apartment | 31°15′18.97″ | 121°33′39.07″ | | residential areas | 3 | 10 | 2 | 3 | 2 | 1 | 0 | 0 | 0 | 0 | |
| 86 | 6 | Gaohang | 20 | Huagao Farmers' Market (No. 1073 Jin Gao Road) | 31°16′55.24″ | 121°36′36.97″ | | farmers' markets | 6 | 5 | 1 | 0 | 0 | 2 | 1 | 1 | 0 | 0 | |
| 87 | 6 | Gaohang | 20 | Jin Gao Restaurant (No. 217 Jin Gao Road) | 31°17′44.59″ | 121°36′26.17″ | | restaurants | 5 | 0 | 0 | 0 | 3 | 2 | 0 | 0 | 0 | 0 | |
| 88 | 6 | Gaohang | 20 | Gaohang Green Park (No. 906 Jinjing Road) | 31°17′14.36″ | 121°36′53.21″ | | parks | 0 | 4 | 3 | 3 | 0 | 0 | 0 | 0 | 0 | 0 | |
| 89 | 6 | Nanhui | 20 | Luchaogang 7th Group (Jiangshan Road) | 30°52′12.38″ | 121°51′34.35″ | | residential areas | 2 | 2 | 0 | 0 | 1 | 3 | 1 | 1 | 0 | 0 | |
| 90 | 6 | Nanhui | 20 | Luchaogang Seafood Market (No. 618 Yugang Road) | 30°51′31.32″ | 121°51′2.52″ | | farmers' markets | 7 | 6 | 3 | 1 | 0 | 0 | 1 | 0 | 0 | 0 | |
| 91 | 6 | Nanhui | 20 | Lingang Zero Point Barbecue City (No. 138, Lane 1889, Jiangshan Road) | 30°52′11.57″ | 121°50′50.52″ | | restaurants | 10 | 1 | 0 | 5 | 2 | 3 | 0 | 0 | 0 | 0 | |
| 92 | 6 | Nanhui | 20 | Luchaogang Park in Nanhui New Town (230 meters east of the intersection of Chaohe Road and Jiangshan Road in Nanhui New Town) | 30°52′8.07″ | 121°51′46.51″ | | parks | 1 | 7 | 1 | 0 | 1 | 0 | 1 | 1 | 0 | 0 | |
| 93 | 6 | Zhuqiao | 20 | Huaxing Village 6 (about 100 meters southwest of Shunchuan Apartment, Xiazhou Road) | 31°12′2.78″ | 121°42′56.64″ | | residential areas | 3 | 3 | 0 | 1 | 1 | 1 | 0 | 0 | 0 | 1 | |
| 94 | 6 | Zhuqiao | 20 | Zhuqiao Trade Market (Hui Shang Apartment Branch) (No. 38 Chuangxin Street) | 31°06′57.51″ | 121°45′33.74″ | | farmers' markets | 3 | 3 | 2 | 2 | 3 | 0 | 1 | 0 | 0 | 0 | |
| 95 | 6 | Zhuqiao | 20 | Tianhe Plaza (No. 666 Baixi Road) | 31°06′59.58″ | 121°45′39.55″ | | restaurants | 12 | 0 | 0 | 0 | 3 | 0 | 0 | 0 | 0 | 0 | |
| 96 | 6 | Zhuqiao | 20 | Haitianhu Park (about 50 meters southeast of the intersection of Weiting Road and Baixi Road (about 50 meters west of Zhuqiao Town Cultural Center)) | 31°06′4.07″ | 121°45′50.62″ | | parks | 1 | 5 | 2 | 2 | 0 | 0 | 1 | 0 | 0 | 0 | |
| 97 | 6 | Sanlin | 20 | Residential Community at No. 334 Lingzhao Road (No. 26, Lane 334 Lingzhao Road) | 31°08′32.17″ | 121°29′49.84″ | | residential areas | 0 | 2 | 0 | 1 | 3 | 2 | 0 | 0 | 0 | 0 | |
| 98 | 6 | Sanlin | 20 | Lingyang Farmers' Market (Room Z-1, 1F, No. 3787 Shangnan Road, H Area, No. 13) | 31°09′6.34″ | 121°30′18.11″ | | farmers' markets | 9 | 2 | 3 | 0 | 2 | 0 | 1 | 0 | 0 | 0 | |
| 99 | 6 | Sanlin | 20 | Legendary Clay Pot (Changqing Road Branch) (No. 2068 Changqing Road) | 31°08′22.31″ | 121°29′49.88″ | | restaurants | 5 | 0 | 0 | 1 | 1 | 1 | 1 | 0 | 0 | 0 | |
| 100 | 6 | Sanlin | 20 | Sanlin Riverside Green Space (140 meters northwest of the intersection of Yanjiang Road and S20 Outer Ring Expressway Auxiliary Road) | 31°07′50.73″ | 121°28′24.94″ | | parks | 0 | 0 | 1 | 1 | 0 | 0 | 0 | 0 | 0 | 0 | |
| 101 | 6 | Tanqiao | 20 | No. 32 Weishan 2nd Village | 31°12′29.25″ | 121°30′48.92″ | | residential areas | 5 | 11 | 0 | 0 | 0 | 1 | 1 | 0 | 0 | 0 | |
| 102 | 6 | Tanqiao | 20 | Pushang Vegetable Market (Nanquan Branch) (No. 1315 Nanquan Road, near Tangqiao Road) | 31°12′36.14″ | 121°31′19.52″ | | farmers' markets | 2 | 4 | 3 | 2 | 0 | 0 | 0 | 0 | 1 | 0 | |
| 103 | 6 | Tanqiao | 20 | Haidilao Hotpot (Paris Spring Pujian Branch) (No. 118 Pujian Road) | 31°12′30.67″ | 121°31′15.17″ | | restaurants | 14 | 0 | 1 | 2 | 2 | 3 | 1 | 0 | 0 | 0 | |
| 104 | 6 | Tanqiao | 20 | Nanpu Square Park (No. 2277 Pudong South Road) | 31°12′13.97″ | 121°30′51.52″ | | parks | 1 | 5 | 3 | 0 | 0 | 1 | 1 | 0 | 0 | 0 | |
| 105 | 6 | Nicheng | 20 | Qingfeng Liyuan (No. 779 Yunhan Road) | 30°54′33.30″ | 121°48′38.59″ | | residential areas | 6 | 3 | 1 | 1 | 3 | 3 | 0 | 0 | 0 | 0 | |
| 106 | 6 | Nicheng | 20 | Fajia Fruit Wholesale (Qiuyu Road Branch) (No. 726 Qiuyu Road) | 30°54′30.85″ | 121°48′58.54″ | | farmers' markets | 8 | 5 | 1 | 2 | 1 | 1 | 1 | 1 | 0 | 0 | |
| 107 | 6 | Nicheng | 20 | Lingang Baolong Plaza (No. 128, Lane 3155 Hongyin Road) | 30°54′22.35″ | 121°48′56.97″ | | restaurants | 6 | 0 | 0 | 0 | 2 | 1 | 0 | 0 | 0 | 0 | |
| 108 | 6 | Nicheng | 20 | Nicheng Riverside Cultural Park (intersection of Pengping Road and Chiyue Road) | 30°54′18.38″ | 121°48′33.39″ | | parks | 0 | 10 | 4 | 1 | 0 | 3 | 0 | 0 | 0 | 0 | |
| 109 | 6 | Huinan | 20 | Dangwan New Village (No. 2 Dangwan Road) | 31°02′39.34″ | 121°45′53.55″ | | residential areas | 4 | 12 | 1 | 0 | 0 | 1 | 1 | 0 | 1 | 1 | |
| 110 | 6 | Huinan | 20 | Dangwan Vegetable Market (Jinghai Road Nanhui Flower and Bird Market) | 31°02′42.04″ | 121°46′3.28″ | | farmers' markets | 1 | 3 | 2 | 2 | 0 | 1 | 0 | 1 | 0 | 0 | |
| 111 | 6 | Huinan | 20 | Guizhou Yellow Beef Hotpot (No. 126 Jinghai Road) | 31°02′40.81″ | 121°46′6.74″ | | restaurants | 5 | 1 | 0 | 1 | 1 | 2 | 1 | 0 | 0 | 0 | |
| 112 | 6 | Huinan | 20 | Nanhui Peach Blossom Village (No. 289 Beimen Road) | 31°03′41.18″ | 121°45′20.02″ | | parks | 0 | 12 | 2 | 3 | 0 | 0 | 0 | 0 | 0 | 0 | |
| 113 | 6 | Caolu | 20 | Jinhai Huacheng Yunxiu Jiayuan (Lane 57, Xiayu Road) | 31°15′21.74″ | 121°41′9.58″ | | residential areas | 7 | 1 | 0 | 0 | 1 | 2 | 1 | 0 | 0 | 0 | |
| 114 | 6 | Caolu | 20 | Yuexing Farmers' Market (Gonglu Vegetable Market Branch) (Room 101-1B, No. 48, Lane 235 Gonghua Road, No. 14-15) | 31°15′53.35″ | 121°41′8.52″ | | farmers' markets | 5 | 2 | 0 | 2 | 1 | 0 | 1 | 2 | 0 | 0 | |
| 115 | 6 | Caolu | 20 | Henan Mixed Noodles and Braised Beef Noodles at Gonglu Xincheng (No. 488 Gonghua Road, Gonghua New Village) | 31°15′33.12″ | 121°41′13.38″ | | restaurants | 8 | 0 | 0 | 3 | 2 | 0 | 1 | 0 | 0 | 0 | |
| 116 | 6 | Caolu | 20 | Firefighting Street Corner Theme Park (northwest of the intersection of Jinhai Road and Lingkong Road) | 31°16′19.67″ | 121°41′12.99″ | | parks | 1 | 3 | 0 | 2 | 0 | 0 | 1 | 3 | 0 | 0 | |
| 117 | 6 | Chuansha | 20 | Xiangchuan Homeland (No. 1-74, Lane 458 Xiangchuan Road) | 31°10′58.12″ | 121°41′42.40″ | | residential areas | 6 | 2 | 0 | 1 | 2 | 0 | 1 | 2 | 0 | 0 | |
| 118 | 6 | Chuansha | 20 | Chuansha Farmers' Market (No. 401 Chuanhuan South Road, Chuanhuang Road) | 31°11′16.80″ | 121°42′19.42″ | | farmers' markets | 6 | 3 | 2 | 4 | 1 | 0 | 1 | 1 | 0 | 0 | |
| 119 | 6 | Chuansha | 20 | Chuansha Commercial Plaza (No. 2, Lane 1136 Miaojing Road, Chuansha New Town) | 31°10′49.97″ | 121°41′35.54″ | | restaurants | 7 | 0 | 0 | 5 | 2 | 1 | 1 | 0 | 0 | 1 | |
| 120 | 6 | Chuansha | 20 | Chuansha Park (No. 437 Chengnan Road) | 31°11′27.06″ | 121°42′3.34″ | | parks | 0 | 0 | 3 | 1 | 0 | 0 | 0 | 0 | 0 | 0 | |
| 121 | 7 | Gaoqiao | 20 | Waigaoqiao Nijin 3D Plaza (Zhangjia Street) | 31°20′45.01″ | 121°34′48.01″ | | residential areas | 12 | 7 | 3 | 1 | 0 | 2 | 0 | 1 | 0 | 1 | |
| 122 | 7 | Gaoqiao | 20 | Gaoqiao Vegetable Market (No. 2 Wangjia Street) | 31°20′53.81″ | 121°34′51.10″ | | farmers' markets | 8 | 3 | 4 | 3 | 3 | 1 | 0 | 1 | 0 | 0 | |
| 123 | 7 | Gaoqiao | 20 | Collection of Books Lamb Restaurant (Huashan Road Branch) (1F, No. 804 Huashan Road) | 31°20′55.77″ | 121°34′45.34″ | | restaurants | 10 | 0 | 0 | 1 | 3 | 2 | 1 | 0 | 0 | 0 | |
| 124 | 7 | Gaoqiao | 20 | Gaoqiao Park (No. 482 Huashan Road) | 31°20′48.41″ | 121°34′21.61″ | | parks | 0 | 1 | 0 | 1 | 0 | 0 | 0 | 1 | 0 | 0 | |
| 125 | 7 | Gaohang | 20 | Longju Apartment | 31°15′18.97″ | 121°33′39.07″ | | residential areas | 7 | 8 | 3 | 2 | 1 | 0 | 1 | 2 | 1 | 0 | |
| 126 | 7 | Gaohang | 20 | Huagao Farmers' Market (No. 1073 Jin Gao Road) | 31°16′55.24″ | 121°36′36.97″ | | farmers' markets | 10 | 0 | 5 | 1 | 4 | 1 | 1 | 2 | 0 | 0 | |
| 127 | 7 | Gaohang | 20 | Jin Gao Restaurant (No. 217 Jin Gao Road) | 31°17′44.59″ | 121°36′26.17″ | | restaurants | 6 | 0 | 0 | 3 | 2 | 1 | 1 | 0 | 0 | 0 | |
| 128 | 7 | Gaohang | 20 | Gaohang Green Park (No. 906 Jinjing Road) | 31°17′14.36″ | 121°36′53.21″ | | parks | 0 | 4 | 0 | 0 | 0 | 0 | 0 | 2 | 0 | 0 | |
| 129 | 7 | Nanhui | 20 | Luchaogang 7th Group (Jiangshan Road) | 30°52′12.38″ | 121°51′34.35″ | | residential areas | 9 | 4 | 3 | 2 | 4 | 0 | 1 | 0 | 1 | 0 | |
| 130 | 7 | Nanhui | 20 | Luchaogang Seafood Market (No. 618 Yugang Road) | 30°51′31.32″ | 121°51′2.52″ | | farmers' markets | 5 | 1 | 3 | 1 | 4 | 1 | 1 | 0 | 1 | 1 | |
| 131 | 7 | Nanhui | 20 | Lingang Zero Point Barbecue City (No. 138, Lane 1889, Jiangshan Road) | 30°52′11.57″ | 121°50′50.52″ | | restaurants | 5 | 1 | 0 | 3 | 1 | 1 | 1 | 0 | 0 | 0 | |
| 132 | 7 | Nanhui | 20 | Luchaogang Park in Nanhui New Town (230 meters east of the intersection of Chaohe Road and Jiangshan Road in Nanhui New Town) | 30°52′8.07″ | 121°51′46.51″ | | parks | 1 | 2 | 2 | 0 | 1 | 0 | 1 | 2 | 0 | 0 | |
| 133 | 7 | Zhuqiao | 20 | Huaxing Village 6 (about 100 meters southwest of Shunchuan Apartment, Xiazhou Road) | 31°12′2.78″ | 121°42′56.64″ | | residential areas | 15 | 11 | 2 | 1 | 2 | 2 | 0 | 0 | 1 | 0 | |
| 134 | 7 | Zhuqiao | 20 | Zhuqiao Trade Market (Hui Shang Apartment Branch) (No. 38 Chuangxin Street) | 31°06′57.51″ | 121°45′33.74″ | | farmers' markets | 8 | 2 | 3 | 1 | 1 | 2 | 0 | 0 | 0 | 1 | |
| 135 | 7 | Zhuqiao | 20 | Tianhe Plaza (No. 666 Baixi Road) | 31°06′59.58″ | 121°45′39.55″ | | restaurants | 8 | 0 | 0 | 1 | 2 | 1 | 1 | 0 | 0 | 1 | |
| 136 | 7 | Zhuqiao | 20 | Haitianhu Park (about 50 meters southeast of the intersection of Weiting Road and Baixi Road (about 50 meters west of Zhuqiao Town Cultural Center)) | 31°06′4.07″ | 121°45′50.62″ | | parks | 0 | 0 | 5 | 3 | 0 | 0 | 0 | 0 | 0 | 0 | |
| 137 | 7 | Sanlin | 20 | Residential Community at No. 334 Lingzhao Road (No. 26, Lane 334 Lingzhao Road) | 31°08′32.17″ | 121°29′49.84″ | | residential areas | 8 | 12 | 4 | 2 | 0 | 1 | 1 | 0 | 0 | 0 | |
| 138 | 7 | Sanlin | 20 | Lingyang Farmers' Market (Room Z-1, 1F, No. 3787 Shangnan Road, H Area, No. 13) | 31°09′6.34″ | 121°30′18.11″ | | farmers' markets | 6 | 1 | 6 | 3 | 2 | 2 | 0 | 1 | 1 | 0 | |
| 139 | 7 | Sanlin | 20 | Legendary Clay Pot (Changqing Road Branch) (No. 2068 Changqing Road) | 31°08′22.31″ | 121°29′49.88″ | | restaurants | 4 | 0 | 0 | 5 | 1 | 2 | 1 | 0 | 0 | 1 | |
| 140 | 7 | Sanlin | 20 | Sanlin Riverside Green Space (140 meters northwest of the intersection of Yanjiang Road and S20 Outer Ring Expressway Auxiliary Road) | 31°07′50.73″ | 121°28′24.94″ | | parks | 0 | 3 | 1 | 5 | 0 | 0 | 0 | 0 | 1 | 0 | |
| 141 | 7 | Tanqiao | 20 | No. 32 Weishan 2nd Village | 31°12′29.25″ | 121°30′48.92″ | | residential areas | 9 | 9 | 3 | 4 | 3 | 0 | 1 | 1 | 0 | 0 | |
| 142 | 7 | Tanqiao | 20 | Pushang Vegetable Market (Nanquan Branch) (No. 1315 Nanquan Road, near Tangqiao Road) | 31°12′36.14″ | 121°31′19.52″ | | farmers' markets | 7 | 2 | 5 | 2 | 0 | 1 | 1 | 0 | 0 | 0 | |
| 143 | 7 | Tanqiao | 20 | Haidilao Hotpot (Paris Spring Pujian Branch) (No. 118 Pujian Road) | 31°12′30.67″ | 121°31′15.17″ | | restaurants | 4 | 0 | 1 | 7 | 2 | 3 | 1 | 0 | 0 | 0 | |
| 144 | 7 | Tanqiao | 20 | Nanpu Square Park (No. 2277 Pudong South Road) | 31°12′13.97″ | 121°30′51.52″ | | parks | 1 | 5 | 3 | 3 | 0 | 1 | 1 | 0 | 0 | 0 | |
| 145 | 7 | Nicheng | 20 | Qingfeng Liyuan (No. 779 Yunhan Road) | 30°54′33.30″ | 121°48′38.59″ | | residential areas | 7 | 7 | 2 | 1 | 5 | 1 | 1 | 0 | 0 | 0 | |
| 146 | 7 | Nicheng | 20 | Fajia Fruit Wholesale (Qiuyu Road Branch) (No. 726 Qiuyu Road) | 30°54′30.85″ | 121°48′58.54″ | | farmers' markets | 6 | 3 | 5 | 5 | 7 | 0 | 1 | 0 | 1 | 0 | |
| 147 | 7 | Nicheng | 20 | Lingang Baolong Plaza (No. 128, Lane 3155 Hongyin Road) | 30°54′22.35″ | 121°48′56.97″ | | restaurants | 5 | 0 | 0 | 4 | 3 | 1 | 1 | 0 | 0 | 0 | |
| 148 | 7 | Nicheng | 20 | Nicheng Riverside Cultural Park (intersection of Pengping Road and Chiyue Road) | 30°54′18.38″ | 121°48′33.39″ | | parks | 0 | 4 | 2 | 5 | 0 | 0 | 0 | 1 | 0 | 0 | |
| 149 | 7 | Huinan | 20 | Dangwan New Village (No. 2 Dangwan Road) | 31°02′39.34″ | 121°45′53.55″ | | residential areas | 5 | 8 | 4 | 0 | 6 | 2 | 1 | 2 | 0 | 1 | |
| 150 | 7 | Huinan | 20 | Dangwan Vegetable Market (Jinghai Road Nanhui Flower and Bird Market) | 31°02′42.04″ | 121°46′3.28″ | | farmers' markets | 3 | 1 | 7 | 5 | 6 | 2 | 1 | 0 | 1 | 0 | |
| 151 | 7 | Huinan | 20 | Guizhou Yellow Beef Hotpot (No. 126 Jinghai Road) | 31°02′40.81″ | 121°46′6.74″ | | restaurants | 11 | 1 | 0 | 7 | 1 | 3 | 0 | 1 | 0 | 0 | |
| 152 | 7 | Huinan | 20 | Nanhui Peach Blossom Village (No. 289 Beimen Road) | 31°03′41.18″ | 121°45′20.02″ | | parks | 1 | 3 | 4 | 0 | 2 | 0 | 1 | 0 | 0 | 0 | |
| 153 | 7 | Caolu | 20 | Jinhai Huacheng Yunxiu Jiayuan (Lane 57, Xiayu Road) | 31°15′21.74″ | 121°41′9.58″ | | residential areas | 9 | 11 | 3 | 2 | 2 | 1 | 1 | 1 | 1 | 0 | |
| 154 | 7 | Caolu | 20 | Yuexing Farmers' Market (Gonglu Vegetable Market Branch) (Room 101-1B, No. 48, Lane 235 Gonghua Road, No. 14-15) | 31°15′53.35″ | 121°41′8.52″ | | farmers' markets | 5 | 3 | 4 | 6 | 1 | 3 | 1 | 0 | 0 | 0 | |
| 155 | 7 | Caolu | 20 | Henan Mixed Noodles and Braised Beef Noodles at Gonglu Xincheng (No. 488 Gonghua Road, Gonghua New Village) | 31°15′33.12″ | 121°41′13.38″ | | restaurants | 7 | 0 | 0 | 3 | 3 | 2 | 1 | 0 | 0 | 1 | |
| 156 | 7 | Caolu | 20 | Firefighting Street Corner Theme Park (northwest of the intersection of Jinhai Road and Lingkong Road) | 31°16′19.67″ | 121°41′12.99″ | | parks | 0 | 2 | 3 | 5 | 0 | 0 | 0 | 0 | 1 | 0 | |
| 157 | 7 | Chuansha | 20 | Xiangchuan Homeland (No. 1-74, Lane 458 Xiangchuan Road) | 31°10′58.12″ | 121°41′42.40″ | | residential areas | 12 | 3 | 3 | 2 | 2 | 3 | 1 | 0 | 0 | 0 | |
| 158 | 7 | Chuansha | 20 | Chuansha Farmers' Market (No. 401 Chuanhuan South Road, Chuanhuang Road) | 31°11′16.80″ | 121°42′19.42″ | | farmers' markets | 9 | 2 | 6 | 2 | 3 | 1 | 0 | 0 | 0 | 0 | |
| 159 | 7 | Chuansha | 20 | Chuansha Commercial Plaza (No. 2, Lane 1136 Miaojing Road, Chuansha New Town) | 31°10′49.97″ | 121°41′35.54″ | | restaurants | 9 | 0 | 1 | 7 | 2 | 2 | 1 | 0 | 1 | 1 | |
| 160 | 7 | Chuansha | 20 | Chuansha Park (No. 437 Chengnan Road) | 31°11′27.06″ | 121°42′3.34″ | | parks | 1 | 6 | 0 | 5 | 0 | 1 | 1 | 2 | 0 | 1 | |
| 161 | 8 | Gaoqiao | 20 | Waigaoqiao Nijin 3D Plaza (Zhangjia Street) | 31°20′45.01″ | 121°34′48.01″ | | residential areas | 10 | 3 | 2 | 7 | 3 | 0 | 1 | 1 | 1 | 0 | |
| 162 | 8 | Gaoqiao | 20 | Gaoqiao Vegetable Market (No. 2 Wangjia Street) | 31°20′53.81″ | 121°34′51.10″ | | farmers' markets | 5 | 1 | 5 | 4 | 3 | 1 | 1 | 2 | 1 | 1 | |
| 163 | 8 | Gaoqiao | 20 | Collection of Books Lamb Restaurant (Huashan Road Branch) (1F, No. 804 Huashan Road) | 31°20′55.77″ | 121°34′45.34″ | | restaurants | 5 | 0 | 0 | 6 | 2 | 1 | 1 | 0 | 0 | 1 | |
| 164 | 8 | Gaoqiao | 20 | Gaoqiao Park (No. 482 Huashan Road) | 31°20′48.41″ | 121°34′21.61″ | | parks | 0 | 0 | 3 | 2 | 0 | 0 | 0 | 0 | 0 | 0 | |
| 165 | 8 | Gaohang | 20 | Longju Apartment | 31°15′18.97″ | 121°33′39.07″ | | residential areas | 6 | 1 | 1 | 1 | 0 | 2 | 2 | 0 | 1 | 0 | |
| 166 | 8 | Gaohang | 20 | Huagao Farmers' Market (No. 1073 Jin Gao Road) | 31°16′55.24″ | 121°36′36.97″ | | farmers' markets | 4 | 2 | 2 | 5 | 1 | 1 | 1 | 0 | 0 | 1 | |
| 167 | 8 | Gaohang | 20 | Jin Gao Restaurant (No. 217 Jin Gao Road) | 31°17′44.59″ | 121°36′26.17″ | | restaurants | 3 | 0 | 1 | 3 | 2 | 2 | 1 | 0 | 0 | 0 | |
| 168 | 8 | Gaohang | 20 | Gaohang Green Park (No. 906 Jinjing Road) | 31°17′14.36″ | 121°36′53.21″ | | parks | 0 | 3 | 1 | 4 | 0 | 1 | 0 | 0 | 1 | 0 | |
| 169 | 8 | Nanhui | 20 | Luchaogang 7th Group (Jiangshan Road) | 30°52′12.38″ | 121°51′34.35″ | | residential areas | 5 | 2 | 2 | 3 | 1 | 1 | 1 | 1 | 0 | 0 | |
| 170 | 8 | Nanhui | 20 | Luchaogang Seafood Market (No. 618 Yugang Road) | 30°51′31.32″ | 121°51′2.52″ | | farmers' markets | 6 | 2 | 3 | 2 | 2 | 2 | 2 | 0 | 0 | 0 | |
| 171 | 8 | Nanhui | 20 | Lingang Zero Point Barbecue City (No. 138, Lane 1889, Jiangshan Road) | 30°52′11.57″ | 121°50′50.52″ | | restaurants | 8 | 0 | 0 | 3 | 2 | 1 | 2 | 0 | 0 | 0 | |
| 172 | 8 | Nanhui | 20 | Luchaogang Park in Nanhui New Town (230 meters east of the intersection of Chaohe Road and Jiangshan Road in Nanhui New Town) | 30°52′8.07″ | 121°51′46.51″ | | parks | 0 | 0 | 2 | 5 | 0 | 0 | 0 | 0 | 0 | 0 | |
| 173 | 8 | Zhuqiao | 20 | Huaxing Village 6 (about 100 meters southwest of Shunchuan Apartment, Xiazhou Road) | 31°12′2.78″ | 121°42′56.64″ | | residential areas | 8 | 1 | 1 | 1 | 0 | 2 | 1 | 1 | 1 | 0 | |
| 174 | 8 | Zhuqiao | 20 | Zhuqiao Trade Market (Hui Shang Apartment Branch) (No. 38 Chuangxin Street) | 31°06′57.51″ | 121°45′33.74″ | | farmers' markets | 7 | 2 | 1 | 4 | 1 | 1 | 1 | 0 | 0 | 1 | |
| 175 | 8 | Zhuqiao | 20 | Tianhe Plaza (No. 666 Baixi Road) | 31°06′59.58″ | 121°45′39.55″ | | restaurants | 5 | 1 | 0 | 2 | 2 | 1 | 1 | 0 | 0 | 0 | |
| 176 | 8 | Zhuqiao | 20 | Haitianhu Park (about 50 meters southeast of the intersection of Weiting Road and Baixi Road (about 50 meters west of Zhuqiao Town Cultural Center)) | 31°06′4.07″ | 121°45′50.62″ | | parks | 0 | 2 | 4 | 3 | 1 | 0 | 0 | 0 | 1 | 0 | |
| 177 | 8 | Sanlin | 20 | Residential Community at No. 334 Lingzhao Road (No. 26, Lane 334 Lingzhao Road) | 31°08′32.17″ | 121°29′49.84″ | | residential areas | 7 | 3 | 2 | 1 | 2 | 2 | 1 | 0 | 0 | 0 | |
| 178 | 8 | Sanlin | 20 | Lingyang Farmers' Market (Room Z-1, 1F, No. 3787 Shangnan Road, H Area, No. 13) | 31°09′6.34″ | 121°30′18.11″ | | farmers' markets | 8 | 1 | 2 | 7 | 3 | 1 | 2 | 0 | 0 | 0 | |
| 179 | 8 | Sanlin | 20 | Legendary Clay Pot (Changqing Road Branch) (No. 2068 Changqing Road) | 31°08′22.31″ | 121°29′49.88″ | | restaurants | 3 | 0 | 0 | 5 | 1 | 2 | 1 | 0 | 0 | 0 | |
| 180 | 8 | Sanlin | 20 | Sanlin Riverside Green Space (140 meters northwest of the intersection of Yanjiang Road and S20 Outer Ring Expressway Auxiliary Road) | 31°07′50.73″ | 121°28′24.94″ | | parks | 0 | 1 | 3 | 2 | 0 | 0 | 0 | 1 | 0 | 0 | |
| 181 | 8 | Tanqiao | 20 | No. 32 Weishan 2nd Village | 31°12′29.25″ | 121°30′48.92″ | | residential areas | 9 | 4 | 3 | 1 | 1 | 1 | 2 | 0 | 0 | 0 | |
| 182 | 8 | Tanqiao | 20 | Pushang Vegetable Market (Nanquan Branch) (No. 1315 Nanquan Road, near Tangqiao Road) | 31°12′36.14″ | 121°31′19.52″ | | farmers' markets | 1 | 2 | 1 | 3 | 4 | 1 | 1 | 0 | 1 | 0 | |
| 183 | 8 | Tanqiao | 20 | Haidilao Hotpot (Paris Spring Pujian Branch) (No. 118 Pujian Road) | 31°12′30.67″ | 121°31′15.17″ | | restaurants | 8 | 0 | 0 | 1 | 2 | 1 | 0 | 0 | 0 | 0 | |
| 184 | 8 | Tanqiao | 20 | Nanpu Square Park (No. 2277 Pudong South Road) | 31°12′13.97″ | 121°30′51.52″ | | parks | 0 | 4 | 1 | 6 | 0 | 0 | 0 | 0 | 1 | 0 | |
| 185 | 8 | Nicheng | 20 | Qingfeng Liyuan (No. 779 Yunhan Road) | 30°54′33.30″ | 121°48′38.59″ | | residential areas | 8 | 1 | 2 | 6 | 3 | 1 | 1 | 1 | 0 | 0 | |
| 186 | 8 | Nicheng | 20 | Fajia Fruit Wholesale (Qiuyu Road Branch) (No. 726 Qiuyu Road) | 30°54′30.85″ | 121°48′58.54″ | | farmers' markets | 4 | 0 | 3 | 5 | 1 | 2 | 2 | 0 | 0 | 0 | |
| 187 | 8 | Nicheng | 20 | Lingang Baolong Plaza (No. 128, Lane 3155 Hongyin Road) | 30°54′22.35″ | 121°48′56.97″ | | restaurants | 4 | 0 | 1 | 4 | 1 | 1 | 1 | 0 | 0 | 0 | |
| 188 | 8 | Nicheng | 20 | Nicheng Riverside Cultural Park (intersection of Pengping Road and Chiyue Road) | 30°54′18.38″ | 121°48′33.39″ | | parks | 1 | 2 | 3 | 3 | 0 | 1 | 0 | 0 | 0 | 0 | |
| 189 | 8 | Huinan | 20 | Dangwan New Village (No. 2 Dangwan Road) | 31°02′39.34″ | 121°45′53.55″ | | residential areas | 6 | 3 | 5 | 2 | 2 | 3 | 0 | 1 | 1 | 0 | |
| 190 | 8 | Huinan | 20 | Dangwan Vegetable Market (Jinghai Road Nanhui Flower and Bird Market) | 31°02′42.04″ | 121°46′3.28″ | | farmers' markets | 8 | 2 | 0 | 5 | 3 | 1 | 2 | 0 | 0 | 0 | |
| 191 | 8 | Huinan | 20 | Guizhou Yellow Beef Hotpot (No. 126 Jinghai Road) | 31°02′40.81″ | 121°46′6.74″ | | restaurants | 8 | 2 | 0 | 4 | 2 | 2 | 1 | 0 | 0 | 0 | |
| 192 | 8 | Huinan | 20 | Nanhui Peach Blossom Village (No. 289 Beimen Road) | 31°03′41.18″ | 121°45′20.02″ | | parks | 0 | 2 | 1 | 2 | 2 | 0 | 0 | 0 | 0 | 0 | |
| 193 | 8 | Caolu | 20 | Jinhai Huacheng Yunxiu Jiayuan (Lane 57, Xiayu Road) | 31°15′21.74″ | 121°41′9.58″ | | residential areas | 13 | 2 | 2 | 2 | 2 | 1 | 0 | 0 | 0 | 0 | |
| 194 | 8 | Caolu | 20 | Yuexing Farmers' Market (Gonglu Vegetable Market Branch) (Room 101-1B, No. 48, Lane 235 Gonghua Road, No. 14-15) | 31°15′53.35″ | 121°41′8.52″ | | farmers' markets | 3 | 2 | 0 | 1 | 2 | 2 | 1 | 0 | 0 | 0 | |
| 195 | 8 | Caolu | 20 | Henan Mixed Noodles and Braised Beef Noodles at Gonglu Xincheng (No. 488 Gonghua Road, Gonghua New Village) | 31°15′33.12″ | 121°41′13.38″ | | restaurants | 6 | 0 | 0 | 2 | 2 | 0 | 2 | 0 | 0 | 1 | |
| 196 | 8 | Caolu | 20 | Firefighting Street Corner Theme Park (northwest of the intersection of Jinhai Road and Lingkong Road) | 31°16′19.67″ | 121°41′12.99″ | | parks | 1 | 4 | 2 | 3 | 0 | 0 | 1 | 2 | 0 | 1 | |
| 197 | 8 | Chuansha | 20 | Xiangchuan Homeland (No. 1-74, Lane 458 Xiangchuan Road) | 31°10′58.12″ | 121°41′42.40″ | | residential areas | 5 | 4 | 2 | 2 | 0 | 2 | 0 | 0 | 0 | 0 | |
| 198 | 8 | Chuansha | 20 | Chuansha Farmers' Market (No. 401 Chuanhuan South Road, Chuanhuang Road) | 31°11′16.80″ | 121°42′19.42″ | | farmers' markets | 1 | 5 | 1 | 4 | 4 | 0 | 1 | 2 | 1 | 0 | |
| 199 | 8 | Chuansha | 20 | Chuansha Commercial Plaza (No. 2, Lane 1136 Miaojing Road, Chuansha New Town) | 31°10′49.97″ | 121°41′35.54″ | | restaurants | 1 | 0 | 0 | 2 | 0 | 1 | 1 | 0 | 0 | 0 | |
| 200 | 8 | Chuansha | 20 | Chuansha Park (No. 437 Chengnan Road) | 31°11′27.06″ | 121°42′3.34″ | | parks | 1 | 3 | 0 | 2 | 0 | 0 | 1 | 0 | 0 | 0 | |
| 201 | 9 | Gaoqiao | 20 | Waigaoqiao Nijin 3D Plaza (Zhangjia Street) | 31°20′45.01″ | 121°34′48.01″ | | residential areas | 5 | 2 | 2 | 1 | 1 | 0 | 0 | 0 | 0 | 0 | |
| 202 | 9 | Gaoqiao | 20 | Gaoqiao Vegetable Market (No. 2 Wangjia Street) | 31°20′53.81″ | 121°34′51.10″ | | farmers' markets | 6 | 1 | 4 | 0 | 2 | 1 | 0 | 0 | 0 | 0 | |
| 203 | 9 | Gaoqiao | 20 | Collection of Books Lamb Restaurant (Huashan Road Branch) (1F, No. 804 Huashan Road) | 31°20′55.77″ | 121°34′45.34″ | | restaurants | 8 | 0 | 0 | 1 | 1 | 2 | 0 | 0 | 0 | 0 | |
| 204 | 9 | Gaoqiao | 20 | Gaoqiao Park (No. 482 Huashan Road) | 31°20′48.41″ | 121°34′21.61″ | | parks | 0 | 1 | 3 | 0 | 0 | 0 | 0 | 0 | 0 | 0 | |
| 205 | 9 | Gaohang | 20 | Longju Apartment | 31°15′18.97″ | 121°33′39.07″ | | residential areas | 9 | 3 | 3 | 3 | 1 | 1 | 1 | 0 | 0 | 0 | |
| 206 | 9 | Gaohang | 20 | Huagao Farmers' Market (No. 1073 Jin Gao Road) | 31°16′55.24″ | 121°36′36.97″ | | farmers' markets | 4 | 2 | 2 | 0 | 1 | 2 | 2 | 0 | 0 | 0 | |
| 207 | 9 | Gaohang | 20 | Jin Gao Restaurant (No. 217 Jin Gao Road) | 31°17′44.59″ | 121°36′26.17″ | | restaurants | 5 | 1 | 0 | 2 | 0 | 2 | 0 | 0 | 0 | 0 | |
| 208 | 9 | Gaohang | 20 | Gaohang Green Park (No. 906 Jinjing Road) | 31°17′14.36″ | 121°36′53.21″ | | parks | 0 | 2 | 0 | 0 | 1 | 0 | 0 | 0 | 0 | 0 | |
| 209 | 9 | Nanhui | 20 | Luchaogang 7th Group (Jiangshan Road) | 30°52′12.38″ | 121°51′34.35″ | | residential areas | 8 | 4 | 2 | 0 | 1 | 0 | 1 | 0 | 0 | 0 | |
| 210 | 9 | Nanhui | 20 | Luchaogang Seafood Market (No. 618 Yugang Road) | 30°51′31.32″ | 121°51′2.52″ | | farmers' markets | 7 | 1 | 3 | 2 | 2 | 1 | 0 | 0 | 0 | 0 | |
| 211 | 9 | Nanhui | 20 | Lingang Zero Point Barbecue City (No. 138, Lane 1889, Jiangshan Road) | 30°52′11.57″ | 121°50′50.52″ | | restaurants | 6 | 0 | 0 | 2 | 2 | 2 | 2 | 0 | 0 | 0 | |
| 212 | 9 | Nanhui | 20 | Luchaogang Park in Nanhui New Town (230 meters east of the intersection of Chaohe Road and Jiangshan Road in Nanhui New Town) | 30°52′8.07″ | 121°51′46.51″ | | parks | 0 | 0 | 2 | 1 | 0 | 0 | 0 | 0 | 0 | 0 | |
| 213 | 9 | Zhuqiao | 20 | Huaxing Village 6 (about 100 meters southwest of Shunchuan Apartment, Xiazhou Road) | 31°12′2.78″ | 121°42′56.64″ | | residential areas | 7 | 1 | 2 | 0 | 0 | 2 | 1 | 0 | 0 | 0 | |
| 214 | 9 | Zhuqiao | 20 | Zhuqiao Trade Market (Hui Shang Apartment Branch) (No. 38 Chuangxin Street) | 31°06′57.51″ | 121°45′33.74″ | | farmers' markets | 3 | 2 | 0 | 2 | 1 | 1 | 0 | 0 | 0 | 0 | |
| 215 | 9 | Zhuqiao | 20 | Tianhe Plaza (No. 666 Baixi Road) | 31°06′59.58″ | 121°45′39.55″ | | restaurants | 4 | 0 | 0 | 1 | 1 | 0 | 1 | 0 | 0 | 0 | |
| 216 | 9 | Zhuqiao | 20 | Haitianhu Park (about 50 meters southeast of the intersection of Weiting Road and Baixi Road (about 50 meters west of Zhuqiao Town Cultural Center)) | 31°06′4.07″ | 121°45′50.62″ | | parks | 0 | 0 | 3 | 2 | 0 | 0 | 0 | 0 | 1 | 0 | |
| 217 | 9 | Sanlin | 20 | Residential Community at No. 334 Lingzhao Road (No. 26, Lane 334 Lingzhao Road) | 31°08′32.17″ | 121°29′49.84″ | | residential areas | 9 | 0 | 1 | 0 | 0 | 2 | 0 | 0 | 0 | 0 | |
| 218 | 9 | Sanlin | 20 | Lingyang Farmers' Market (Room Z-1, 1F, No. 3787 Shangnan Road, H Area, No. 13) | 31°09′6.34″ | 121°30′18.11″ | | farmers' markets | 7 | 2 | 2 | 2 | 0 | 1 | 0 | 0 | 0 | 0 | |
| 219 | 9 | Sanlin | 20 | Legendary Clay Pot (Changqing Road Branch) (No. 2068 Changqing Road) | 31°08′22.31″ | 121°29′49.88″ | | restaurants | 6 | 0 | 0 | 3 | 2 | 2 | 2 | 0 | 0 | 0 | |
| 220 | 9 | Sanlin | 20 | Sanlin Riverside Green Space (140 meters northwest of the intersection of Yanjiang Road and S20 Outer Ring Expressway Auxiliary Road) | 31°07′50.73″ | 121°28′24.94″ | | parks | 0 | 3 | 5 | 0 | 0 | 0 | 1 | 0 | 0 | 0 | |
| 221 | 9 | Tanqiao | 20 | No. 32 Weishan 2nd Village | 31°12′29.25″ | 121°30′48.92″ | | residential areas | 14 | 2 | 2 | 1 | 2 | 1 | 0 | 0 | 0 | 0 | |
| 222 | 9 | Tanqiao | 20 | Pushang Vegetable Market (Nanquan Branch) (No. 1315 Nanquan Road, near Tangqiao Road) | 31°12′36.14″ | 121°31′19.52″ | | farmers' markets | 6 | 1 | 1 | 5 | 2 | 1 | 0 | 1 | 1 | 0 | |
| 223 | 9 | Tanqiao | 20 | Haidilao Hotpot (Paris Spring Pujian Branch) (No. 118 Pujian Road) | 31°12′30.67″ | 121°31′15.17″ | | restaurants | 8 | 0 | 1 | 0 | 1 | 1 | 0 | 0 | 0 | 0 | |
| 224 | 9 | Tanqiao | 20 | Nanpu Square Park (No. 2277 Pudong South Road) | 31°12′13.97″ | 121°30′51.52″ | | parks | 0 | 1 | 0 | 2 | 0 | 1 | 0 | 0 | 0 | 0 | |
| 225 | 9 | Nicheng | 20 | Qingfeng Liyuan (No. 779 Yunhan Road) | 30°54′33.30″ | 121°48′38.59″ | | residential areas | 9 | 3 | 2 | 0 | 1 | 0 | 0 | 0 | 0 | 0 | |
| 226 | 9 | Nicheng | 20 | Fajia Fruit Wholesale (Qiuyu Road Branch) (No. 726 Qiuyu Road) | 30°54′30.85″ | 121°48′58.54″ | | farmers' markets | 7 | 2 | 1 | 3 | 1 | 1 | 0 | 0 | 0 | 0 | |
| 227 | 9 | Nicheng | 20 | Lingang Baolong Plaza (No. 128, Lane 3155 Hongyin Road) | 30°54′22.35″ | 121°48′56.97″ | | restaurants | 5 | 0 | 0 | 3 | 0 | 1 | 2 | 0 | 0 | 0 | |
| 228 | 9 | Nicheng | 20 | Nicheng Riverside Cultural Park (intersection of Pengping Road and Chiyue Road) | 30°54′18.38″ | 121°48′33.39″ | | parks | 0 | 4 | 0 | 1 | 0 | 0 | 0 | 0 | 0 | 0 | |
| 229 | 9 | Huinan | 20 | Dangwan New Village (No. 2 Dangwan Road) | 31°02′39.34″ | 121°45′53.55″ | | residential areas | 6 | 1 | 0 | 0 | 2 | 0 | 0 | 0 | 0 | 0 | |
| 230 | 9 | Huinan | 20 | Dangwan Vegetable Market (Jinghai Road Nanhui Flower and Bird Market) | 31°02′42.04″ | 121°46′3.28″ | | farmers' markets | 2 | 3 | 2 | 2 | 1 | 0 | 0 | 0 | 0 | 0 | |
| 231 | 9 | Huinan | 20 | Guizhou Yellow Beef Hotpot (No. 126 Jinghai Road) | 31°02′40.81″ | 121°46′6.74″ | | restaurants | 7 | 1 | 0 | 0 | 2 | 1 | 0 | 0 | 0 | 0 | |
| 232 | 9 | Huinan | 20 | Nanhui Peach Blossom Village (No. 289 Beimen Road) | 31°03′41.18″ | 121°45′20.02″ | | parks | 1 | 3 | 2 | 3 | 1 | 0 | 1 | 0 | 0 | 0 | |
| 233 | 9 | Caolu | 20 | Jinhai Huacheng Yunxiu Jiayuan (Lane 57, Xiayu Road) | 31°15′21.74″ | 121°41′9.58″ | | residential areas | 5 | 1 | 3 | 0 | 3 | 1 | 0 | 0 | 0 | 0 | |
| 234 | 9 | Caolu | 20 | Yuexing Farmers' Market (Gonglu Vegetable Market Branch) (Room 101-1B, No. 48, Lane 235 Gonghua Road, No. 14-15) | 31°15′53.35″ | 121°41′8.52″ | | farmers' markets | 6 | 1 | 2 | 1 | 1 | 2 | 0 | 0 | 0 | 0 | |
| 235 | 9 | Caolu | 20 | Henan Mixed Noodles and Braised Beef Noodles at Gonglu Xincheng (No. 488 Gonghua Road, Gonghua New Village) | 31°15′33.12″ | 121°41′13.38″ | | restaurants | 6 | 0 | 0 | 2 | 1 | 1 | 0 | 0 | 0 | 0 | |
| 236 | 9 | Caolu | 20 | Firefighting Street Corner Theme Park (northwest of the intersection of Jinhai Road and Lingkong Road) | 31°16′19.67″ | 121°41′12.99″ | | parks | 0 | 0 | 3 | 0 | 0 | 0 | 0 | 0 | 0 | 0 | |
| 237 | 9 | Chuansha | 20 | Xiangchuan Homeland (No. 1-74, Lane 458 Xiangchuan Road) | 31°10′58.12″ | 121°41′42.40″ | | residential areas | 6 | 3 | 3 | 0 | 0 | 2 | 0 | 0 | 0 | 0 | |
| 238 | 9 | Chuansha | 20 | Chuansha Farmers' Market (No. 401 Chuanhuan South Road, Chuanhuang Road) | 31°11′16.80″ | 121°42′19.42″ | | farmers' markets | 8 | 3 | 1 | 2 | 2 | 1 | 0 | 0 | 1 | 0 | |
| 239 | 9 | Chuansha | 20 | Chuansha Commercial Plaza (No. 2, Lane 1136 Miaojing Road, Chuansha New Town) | 31°10′49.97″ | 121°41′35.54″ | | restaurants | 11 | 0 | 1 | 4 | 2 | 1 | 0 | 0 | 0 | 0 | |
| 240 | 9 | Chuansha | 20 | Chuansha Park (No. 437 Chengnan Road) | 31°11′27.06″ | 121°42′3.34″ | | parks | 1 | 6 | 2 | 0 | 0 | 1 | 0 | 0 | 0 | 0 | |
| 241 | 10 | Gaoqiao | 20 | Waigaoqiao Nijin 3D Plaza (Zhangjia Street) | 31°20′45.01″ | 121°34′48.01″ | | residential areas | 4 | 1 | 2 | 2 | 0 | 0 | 0 | 0 | 0 | 0 | |
| 242 | 10 | Gaoqiao | 20 | Gaoqiao Vegetable Market (No. 2 Wangjia Street) | 31°20′53.81″ | 121°34′51.10″ | | farmers' markets | 8 | 3 | 2 | 2 | 1 | 0 | 0 | 0 | 0 | 0 | |
| 243 | 10 | Gaoqiao | 20 | Collection of Books Lamb Restaurant (Huashan Road Branch) (1F, No. 804 Huashan Road) | 31°20′55.77″ | 121°34′45.34″ | | restaurants | 8 | 0 | 0 | 0 | 0 | 0 | 0 | 0 | 0 | 0 | |
| 244 | 10 | Gaoqiao | 20 | Gaoqiao Park (No. 482 Huashan Road) | 31°20′48.41″ | 121°34′21.61″ | | parks | 0 | 3 | 3 | 2 | 0 | 0 | 0 | 0 | 0 | 0 | |
| 245 | 10 | Gaohang | 20 | Longju Apartment | 31°15′18.97″ | 121°33′39.07″ | | residential areas | 7 | 2 | 1 | 1 | 0 | 0 | 0 | 0 | 0 | 0 | |
| 246 | 10 | Gaohang | 20 | Huagao Farmers' Market (No. 1073 Jin Gao Road) | 31°16′55.24″ | 121°36′36.97″ | | farmers' markets | 6 | 2 | 3 | 1 | 0 | 0 | 0 | 0 | 0 | 0 | |
| 247 | 10 | Gaohang | 20 | Jin Gao Restaurant (No. 217 Jin Gao Road) | 31°17′44.59″ | 121°36′26.17″ | | restaurants | 15 | 0 | 0 | 0 | 1 | 0 | 0 | 0 | 0 | 0 | |
| 248 | 10 | Gaohang | 20 | Gaohang Green Park (No. 906 Jinjing Road) | 31°17′14.36″ | 121°36′53.21″ | | parks | 0 | 0 | 0 | 2 | 0 | 0 | 0 | 0 | 0 | 0 | |
| 249 | 10 | Nanhui | 20 | Luchaogang 7th Group (Jiangshan Road) | 30°52′12.38″ | 121°51′34.35″ | | residential areas | 12 | 2 | 2 | 0 | 0 | 0 | 0 | 0 | 0 | 0 | |
| 250 | 10 | Nanhui | 20 | Luchaogang Seafood Market (No. 618 Yugang Road) | 30°51′31.32″ | 121°51′2.52″ | | farmers' markets | 5 | 2 | 2 | 1 | 0 | 0 | 0 | 0 | 0 | 0 | |
| 251 | 10 | Nanhui | 20 | Lingang Zero Point Barbecue City (No. 138, Lane 1889, Jiangshan Road) | 30°52′11.57″ | 121°50′50.52″ | | restaurants | 4 | 0 | 0 | 2 | 1 | 0 | 0 | 0 | 0 | 0 | |
| 252 | 10 | Nanhui | 20 | Luchaogang Park in Nanhui New Town (230 meters east of the intersection of Chaohe Road and Jiangshan Road in Nanhui New Town) | 30°52′8.07″ | 121°51′46.51″ | | parks | 0 | 2 | 3 | 0 | 0 | 0 | 0 | 0 | 0 | 0 | |
| 253 | 10 | Zhuqiao | 20 | Huaxing Village 6 (about 100 meters southwest of Shunchuan Apartment, Xiazhou Road) | 31°12′2.78″ | 121°42′56.64″ | | residential areas | 8 | 0 | 3 | 0 | 0 | 0 | 0 | 0 | 0 | 0 | |
| 254 | 10 | Zhuqiao | 20 | Zhuqiao Trade Market (Hui Shang Apartment Branch) (No. 38 Chuangxin Street) | 31°06′57.51″ | 121°45′33.74″ | | farmers' markets | 3 | 3 | 1 | 0 | 0 | 0 | 0 | 0 | 0 | 0 | |
| 255 | 10 | Zhuqiao | 20 | Tianhe Plaza (No. 666 Baixi Road) | 31°06′59.58″ | 121°45′39.55″ | | restaurants | 6 | 0 | 0 | 0 | 0 | 0 | 0 | 0 | 0 | 0 | |
| 256 | 10 | Zhuqiao | 20 | Haitianhu Park (about 50 meters southeast of the intersection of Weiting Road and Baixi Road (about 50 meters west of Zhuqiao Town Cultural Center)) | 31°06′4.07″ | 121°45′50.62″ | | parks | 0 | 0 | 1 | 1 | 0 | 0 | 0 | 0 | 0 | 0 | |
| 257 | 10 | Sanlin | 20 | Residential Community at No. 334 Lingzhao Road (No. 26, Lane 334 Lingzhao Road) | 31°08′32.17″ | 121°29′49.84″ | | residential areas | 7 | 2 | 1 | 0 | 0 | 0 | 0 | 0 | 0 | 0 | |
| 258 | 10 | Sanlin | 20 | Lingyang Farmers' Market (Room Z-1, 1F, No. 3787 Shangnan Road, H Area, No. 13) | 31°09′6.34″ | 121°30′18.11″ | | farmers' markets | 8 | 2 | 2 | 2 | 1 | 0 | 0 | 0 | 0 | 0 | |
| 259 | 10 | Sanlin | 20 | Legendary Clay Pot (Changqing Road Branch) (No. 2068 Changqing Road) | 31°08′22.31″ | 121°29′49.88″ | | restaurants | 7 | 0 | 0 | 3 | 1 | 0 | 0 | 0 | 0 | 0 | |
| 260 | 10 | Sanlin | 20 | Sanlin Riverside Green Space (140 meters northwest of the intersection of Yanjiang Road and S20 Outer Ring Expressway Auxiliary Road) | 31°07′50.73″ | 121°28′24.94″ | | parks | 0 | 3 | 1 | 2 | 0 | 0 | 0 | 0 | 0 | 0 | |
| 261 | 10 | Tanqiao | 20 | No. 32 Weishan 2nd Village | 31°12′29.25″ | 121°30′48.92″ | | residential areas | 9 | 2 | 0 | 0 | 1 | 0 | 0 | 0 | 0 | 0 | |
| 262 | 10 | Tanqiao | 20 | Pushang Vegetable Market (Nanquan Branch) (No. 1315 Nanquan Road, near Tangqiao Road) | 31°12′36.14″ | 121°31′19.52″ | | farmers' markets | 6 | 1 | 1 | 1 | 1 | 0 | 0 | 0 | 0 | 0 | |
| 263 | 10 | Tanqiao | 20 | Haidilao Hotpot (Paris Spring Pujian Branch) (No. 118 Pujian Road) | 31°12′30.67″ | 121°31′15.17″ | | restaurants | 5 | 0 | 0 | 2 | 1 | 0 | 0 | 0 | 0 | 0 | |
| 264 | 10 | Tanqiao | 20 | Nanpu Square Park (No. 2277 Pudong South Road) | 31°12′13.97″ | 121°30′51.52″ | | parks | 0 | 5 | 3 | 2 | 0 | 0 | 0 | 0 | 0 | 0 | |
| 265 | 10 | Nicheng | 20 | Qingfeng Liyuan (No. 779 Yunhan Road) | 30°54′33.30″ | 121°48′38.59″ | | residential areas | 4 | 2 | 3 | 0 | 0 | 0 | 0 | 0 | 0 | 0 | |
| 266 | 10 | Nicheng | 20 | Fajia Fruit Wholesale (Qiuyu Road Branch) (No. 726 Qiuyu Road) | 30°54′30.85″ | 121°48′58.54″ | | farmers' markets | 5 | 3 | 2 | 1 | 1 | 0 | 0 | 0 | 0 | 0 | |
| 267 | 10 | Nicheng | 20 | Lingang Baolong Plaza (No. 128, Lane 3155 Hongyin Road) | 30°54′22.35″ | 121°48′56.97″ | | restaurants | 6 | 0 | 0 | 0 | 0 | 0 | 0 | 0 | 0 | 0 | |
| 268 | 10 | Nicheng | 20 | Nicheng Riverside Cultural Park (intersection of Pengping Road and Chiyue Road) | 30°54′18.38″ | 121°48′33.39″ | | parks | 0 | 4 | 5 | 0 | 0 | 0 | 0 | 0 | 0 | 0 | |
| 269 | 10 | Huinan | 20 | Dangwan New Village (No. 2 Dangwan Road) | 31°02′39.34″ | 121°45′53.55″ | | residential areas | 5 | 1 | 2 | 0 | 0 | 0 | 0 | 0 | 0 | 0 | |
| 270 | 10 | Huinan | 20 | Dangwan Vegetable Market (Jinghai Road Nanhui Flower and Bird Market) | 31°02′42.04″ | 121°46′3.28″ | | farmers' markets | 7 | 2 | 1 | 1 | 1 | 0 | 0 | 0 | 0 | 0 | |
| 271 | 10 | Huinan | 20 | Guizhou Yellow Beef Hotpot (No. 126 Jinghai Road) | 31°02′40.81″ | 121°46′6.74″ | | restaurants | 14 | 0 | 0 | 1 | 0 | 0 | 0 | 0 | 0 | 0 | |
| 272 | 10 | Huinan | 20 | Nanhui Peach Blossom Village (No. 289 Beimen Road) | 31°03′41.18″ | 121°45′20.02″ | | parks | 0 | 2 | 2 | 0 | 0 | 0 | 0 | 0 | 0 | 0 | |
| 273 | 10 | Caolu | 20 | Jinhai Huacheng Yunxiu Jiayuan (Lane 57, Xiayu Road) | 31°15′21.74″ | 121°41′9.58″ | | residential areas | 7 | 2 | 2 | 1 | 1 | 0 | 0 | 0 | 0 | 0 | |
| 274 | 10 | Caolu | 20 | Yuexing Farmers' Market (Gonglu Vegetable Market Branch) (Room 101-1B, No. 48, Lane 235 Gonghua Road, No. 14-15) | 31°15′53.35″ | 121°41′8.52″ | | farmers' markets | 4 | 2 | 1 | 0 | 0 | 0 | 0 | 0 | 0 | 0 | |
| 275 | 10 | Caolu | 20 | Henan Mixed Noodles and Braised Beef Noodles at Gonglu Xincheng (No. 488 Gonghua Road, Gonghua New Village) | 31°15′33.12″ | 121°41′13.38″ | | restaurants | 16 | 0 | 0 | 1 | 1 | 0 | 0 | 0 | 0 | 0 | |
| 276 | 10 | Caolu | 20 | Firefighting Street Corner Theme Park (northwest of the intersection of Jinhai Road and Lingkong Road) | 31°16′19.67″ | 121°41′12.99″ | | parks | 0 | 0 | 0 | 1 | 0 | 0 | 0 | 0 | 0 | 0 | |
| 277 | 10 | Chuansha | 20 | Xiangchuan Homeland (No. 1-74, Lane 458 Xiangchuan Road) | 31°10′58.12″ | 121°41′42.40″ | | residential areas | 6 | 3 | 2 | 1 | 0 | 0 | 0 | 0 | 0 | 0 | |
| 278 | 10 | Chuansha | 20 | Chuansha Farmers' Market (No. 401 Chuanhuan South Road, Chuanhuang Road) | 31°11′16.80″ | 121°42′19.42″ | | farmers' markets | 6 | 1 | 3 | 0 | 0 | 0 | 0 | 0 | 0 | 0 | |
| 279 | 10 | Chuansha | 20 | Chuansha Commercial Plaza (No. 2, Lane 1136 Miaojing Road, Chuansha New Town) | 31°10′49.97″ | 121°41′35.54″ | | restaurants | 5 | 0 | 0 | 0 | 1 | 0 | 0 | 0 | 0 | 0 | |
| 280 | 10 | Chuansha | 20 | Chuansha Park (No. 437 Chengnan Road) | 31°11′27.06″ | 121°42′3.34″ | | parks | 0 | 1 | 2 | 1 | 0 | 0 | 0 | 0 | 0 | 0 | |
| 281 | 11 | Gaoqiao | 20 | Waigaoqiao Nijin 3D Plaza (Zhangjia Street) | 31°20′45.01″ | 121°34′48.01″ | | residential areas | 6 | 3 | 2 | 0 | 1 | 0 | 0 | 0 | 0 | 0 | |
| 282 | 11 | Gaoqiao | 20 | Gaoqiao Vegetable Market (No. 2 Wangjia Street) | 31°20′53.81″ | 121°34′51.10″ | | farmers' markets | 7 | 2 | 2 | 1 | 0 | 0 | 0 | 0 | 0 | 0 | |
| 283 | 11 | Gaoqiao | 20 | Collection of Books Lamb Restaurant (Huashan Road Branch) (1F, No. 804 Huashan Road) | 31°20′55.77″ | 121°34′45.34″ | | restaurants | 6 | 0 | 0 | 1 | 1 | 0 | 0 | 0 | 0 | 0 | |
| 284 | 11 | Gaoqiao | 20 | Gaoqiao Park (No. 482 Huashan Road) | 31°20′48.41″ | 121°34′21.61″ | | parks | 0 | 0 | 2 | 2 | 0 | 0 | 0 | 0 | 0 | 0 | |
| 285 | 11 | Gaohang | 20 | Longju Apartment | 31°15′18.97″ | 121°33′39.07″ | | residential areas | 9 | 2 | 0 | 0 | 0 | 0 | 0 | 0 | 0 | 0 | |
| 286 | 11 | Gaohang | 20 | Huagao Farmers' Market (No. 1073 Jin Gao Road) | 31°16′55.24″ | 121°36′36.97″ | | farmers' markets | 4 | 2 | 2 | 2 | 1 | 0 | 0 | 0 | 0 | 0 | |
| 287 | 11 | Gaohang | 20 | Jin Gao Restaurant (No. 217 Jin Gao Road) | 31°17′44.59″ | 121°36′26.17″ | | restaurants | 22 | 0 | 0 | 2 | 1 | 0 | 0 | 0 | 0 | 0 | |
| 288 | 11 | Gaohang | 20 | Gaohang Green Park (No. 906 Jinjing Road) | 31°17′14.36″ | 121°36′53.21″ | | parks | 0 | 3 | 0 | 0 | 0 | 0 | 0 | 0 | 0 | 0 | |
| 289 | 11 | Nanhui | 20 | Luchaogang 7th Group (Jiangshan Road) | 30°52′12.38″ | 121°51′34.35″ | | residential areas | 7 | 3 | 2 | 0 | 1 | 0 | 0 | 0 | 0 | 0 | |
| 290 | 11 | Nanhui | 20 | Luchaogang Seafood Market (No. 618 Yugang Road) | 30°51′31.32″ | 121°51′2.52″ | | farmers' markets | 13 | 3 | 2 | 2 | 0 | 0 | 0 | 0 | 0 | 0 | |
| 291 | 11 | Nanhui | 20 | Lingang Zero Point Barbecue City (No. 138, Lane 1889, Jiangshan Road) | 30°52′11.57″ | 121°50′50.52″ | | restaurants | 7 | 0 | 0 | 1 | 1 | 0 | 0 | 0 | 0 | 0 | |
| 292 | 11 | Nanhui | 20 | Luchaogang Park in Nanhui New Town (230 meters east of the intersection of Chaohe Road and Jiangshan Road in Nanhui New Town) | 30°52′8.07″ | 121°51′46.51″ | | parks | 0 | 4 | 1 | 1 | 0 | 0 | 0 | 0 | 0 | 0 | |
| 293 | 11 | Zhuqiao | 20 | Huaxing Village 6 (about 100 meters southwest of Shunchuan Apartment, Xiazhou Road) | 31°12′2.78″ | 121°42′56.64″ | | residential areas | 10 | 2 | 3 | 0 | 1 | 0 | 0 | 0 | 0 | 0 | |
| 294 | 11 | Zhuqiao | 20 | Zhuqiao Trade Market (Hui Shang Apartment Branch) (No. 38 Chuangxin Street) | 31°06′57.51″ | 121°45′33.74″ | | farmers' markets | 8 | 2 | 3 | 0 | 1 | 0 | 0 | 0 | 0 | 0 | |
| 295 | 11 | Zhuqiao | 20 | Tianhe Plaza (No. 666 Baixi Road) | 31°06′59.58″ | 121°45′39.55″ | | restaurants | 5 | 0 | 0 | 0 | 0 | 0 | 0 | 0 | 0 | 0 | |
| 296 | 11 | Zhuqiao | 20 | Haitianhu Park (about 50 meters southeast of the intersection of Weiting Road and Baixi Road (about 50 meters west of Zhuqiao Town Cultural Center)) | 31°06′4.07″ | 121°45′50.62″ | | parks | 0 | 3 | 2 | 1 | 0 | 0 | 0 | 0 | 0 | 0 | |
| 297 | 11 | Sanlin | 20 | Residential Community at No. 334 Lingzhao Road (No. 26, Lane 334 Lingzhao Road) | 31°08′32.17″ | 121°29′49.84″ | | residential areas | 8 | 1 | 1 | 0 | 1 | 0 | 0 | 0 | 0 | 0 | |
| 298 | 11 | Sanlin | 20 | Lingyang Farmers' Market (Room Z-1, 1F, No. 3787 Shangnan Road, H Area, No. 13) | 31°09′6.34″ | 121°30′18.11″ | | farmers' markets | 5 | 3 | 1 | 1 | 0 | 0 | 0 | 0 | 0 | 0 | |
| 299 | 11 | Sanlin | 20 | Legendary Clay Pot (Changqing Road Branch) (No. 2068 Changqing Road) | 31°08′22.31″ | 121°29′49.88″ | | restaurants | 16 | 0 | 0 | 0 | 0 | 0 | 0 | 0 | 0 | 0 | |
| 300 | 11 | Sanlin | 20 | Sanlin Riverside Green Space (140 meters northwest of the intersection of Yanjiang Road and S20 Outer Ring Expressway Auxiliary Road) | 31°07′50.73″ | 121°28′24.94″ | | parks | 0 | 0 | 3 | 1 | 0 | 0 | 0 | 0 | 0 | 0 | |
| 301 | 11 | Tanqiao | 20 | No. 32 Weishan 2nd Village | 31°12′29.25″ | 121°30′48.92″ | | residential areas | 5 | 2 | 3 | 1 | 0 | 0 | 0 | 0 | 0 | 0 | |
| 302 | 11 | Tanqiao | 20 | Pushang Vegetable Market (Nanquan Branch) (No. 1315 Nanquan Road, near Tangqiao Road) | 31°12′36.14″ | 121°31′19.52″ | | farmers' markets | 12 | 2 | 2 | 0 | 0 | 0 | 0 | 0 | 0 | 0 | |
| 303 | 11 | Tanqiao | 20 | Haidilao Hotpot (Paris Spring Pujian Branch) (No. 118 Pujian Road) | 31°12′30.67″ | 121°31′15.17″ | | restaurants | 3 | 0 | 0 | 2 | 0 | 0 | 0 | 0 | 0 | 0 | |
| 304 | 11 | Tanqiao | 20 | Nanpu Square Park (No. 2277 Pudong South Road) | 31°12′13.97″ | 121°30′51.52″ | | parks | 0 | 2 | 3 | 1 | 0 | 0 | 0 | 0 | 0 | 0 | |
| 305 | 11 | Nicheng | 20 | Qingfeng Liyuan (No. 779 Yunhan Road) | 30°54′33.30″ | 121°48′38.59″ | | residential areas | 6 | 2 | 4 | 0 | 0 | 0 | 0 | 0 | 0 | 0 | |
| 306 | 11 | Nicheng | 20 | Fajia Fruit Wholesale (Qiuyu Road Branch) (No. 726 Qiuyu Road) | 30°54′30.85″ | 121°48′58.54″ | | farmers' markets | 19 | 1 | 2 | 2 | 0 | 0 | 0 | 0 | 0 | 0 | |
| 307 | 11 | Nicheng | 20 | Lingang Baolong Plaza (No. 128, Lane 3155 Hongyin Road) | 30°54′22.35″ | 121°48′56.97″ | | restaurants | 15 | 0 | 0 | 0 | 0 | 0 | 0 | 0 | 0 | 0 | |
| 308 | 11 | Nicheng | 20 | Nicheng Riverside Cultural Park (intersection of Pengping Road and Chiyue Road) | 30°54′18.38″ | 121°48′33.39″ | | parks | 0 | 0 | 4 | 0 | 0 | 0 | 0 | 0 | 0 | 0 | |
| 309 | 11 | Huinan | 20 | Dangwan New Village (No. 2 Dangwan Road) | 31°02′39.34″ | 121°45′53.55″ | | residential areas | 8 | 2 | 1 | 0 | 0 | 0 | 0 | 0 | 0 | 0 | |
| 310 | 11 | Huinan | 20 | Dangwan Vegetable Market (Jinghai Road Nanhui Flower and Bird Market) | 31°02′42.04″ | 121°46′3.28″ | | farmers' markets | 6 | 2 | 1 | 0 | 0 | 0 | 0 | 0 | 0 | 0 | |
| 311 | 11 | Huinan | 20 | Guizhou Yellow Beef Hotpot (No. 126 Jinghai Road) | 31°02′40.81″ | 121°46′6.74″ | | restaurants | 7 | 0 | 0 | 2 | 0 | 0 | 0 | 0 | 0 | 0 | |
| 312 | 11 | Huinan | 20 | Nanhui Peach Blossom Village (No. 289 Beimen Road) | 31°03′41.18″ | 121°45′20.02″ | | parks | 0 | 1 | 1 | 3 | 0 | 0 | 0 | 0 | 0 | 0 | |
| 313 | 11 | Caolu | 20 | Jinhai Huacheng Yunxiu Jiayuan (Lane 57, Xiayu Road) | 31°15′21.74″ | 121°41′9.58″ | | residential areas | 7 | 3 | 2 | 0 | 0 | 0 | 0 | 0 | 0 | 0 | |
| 314 | 11 | Caolu | 20 | Yuexing Farmers' Market (Gonglu Vegetable Market Branch) (Room 101-1B, No. 48, Lane 235 Gonghua Road, No. 14-15) | 31°15′53.35″ | 121°41′8.52″ | | farmers' markets | 5 | 2 | 2 | 0 | 0 | 0 | 0 | 0 | 0 | 0 | |
| 315 | 11 | Caolu | 20 | Henan Mixed Noodles and Braised Beef Noodles at Gonglu Xincheng (No. 488 Gonghua Road, Gonghua New Village) | 31°15′33.12″ | 121°41′13.38″ | | restaurants | 6 | 0 | 0 | 1 | 0 | 0 | 0 | 0 | 0 | 0 | |
| 316 | 11 | Caolu | 20 | Firefighting Street Corner Theme Park (northwest of the intersection of Jinhai Road and Lingkong Road) | 31°16′19.67″ | 121°41′12.99″ | | parks | 0 | 4 | 5 | 0 | 0 | 0 | 0 | 0 | 0 | 0 | |
| 317 | 11 | Chuansha | 20 | Xiangchuan Homeland (No. 1-74, Lane 458 Xiangchuan Road) | 31°10′58.12″ | 121°41′42.40″ | | residential areas | 10 | 2 | 3 | 0 | 0 | 0 | 0 | 0 | 0 | 0 | |
| 318 | 11 | Chuansha | 20 | Chuansha Farmers' Market (No. 401 Chuanhuan South Road, Chuanhuang Road) | 31°11′16.80″ | 121°42′19.42″ | | farmers' markets | 6 | 4 | 3 | 0 | 0 | 0 | 0 | 0 | 0 | 0 | |
| 319 | 11 | Chuansha | 20 | Chuansha Commercial Plaza (No. 2, Lane 1136 Miaojing Road, Chuansha New Town) | 31°10′49.97″ | 121°41′35.54″ | | restaurants | 8 | 0 | 0 | 0 | 0 | 0 | 0 | 0 | 0 | 0 | |
| 320 | 11 | Chuansha | 20 | Chuansha Park (No. 437 Chengnan Road) | 31°11′27.06″ | 121°42′3.34″ | | parks | 0 | 3 | 4 | 0 | 0 | 0 | 0 | 0 | 0 | 0 | |
|  |  |  |  |  |  |  | |  |  |  |  |  |  |  |  |  |  |  | |
